# An *in vitro* intestinal model captures immunomodulatory properties of the microbiota in inflammation

**DOI:** 10.1080/19490976.2022.2039002

**Published:** 2022-03-22

**Authors:** Jaclyn Y. Lock, Mariaelena Caboni, Philip Strandwitz, Madeleine Morrissette, Kevin DiBona, Brian A. Joughin, Kim Lewis, Rebecca L. Carrier

**Affiliations:** a Department of Bioengineering, Northeastern University, Boston, Massachusetts, USA; b Antimicrobial Discovery Center, Department of Biology, Northeastern University, Boston, Massachusetts, USA; c Department of Biochemistry, Northeastern University, Boston, Massachusetts, USA; d The Koch Institute for Integrative Cancer Research at Mit and the Department of Biological Engineering, Massachusetts Institute of Technology, Cambridge, Massacusetts, USA; e Department of Chemical Engineering, Northeastern University, Boston, Massachusetts, USA; f Department of Biology, Northeastern University, Boston, Massachusetts, USA

**Keywords:** Gut microbiome, inflammation, intestinal model, innate immune cells, gut simulator, anaerobic respiration, *Enterobacteriaceae*, endotoxin

## Abstract

Considerable effort has been put forth to understand mechanisms by which the microbiota modulates and responds to inflammation. Here, we explored whether oxidation metabolites produced by the host during inflammation, sodium nitrate and trimethylamine oxide, impact the composition of a human stool bacterial population in a gut simulator. We then assessed whether an immune-competent *in vitro* intestinal model responded differently to spent medium from bacteria exposed to these cues compared to spent medium from a control bacterial population. The host-derived oxidation products were found to decrease levels of *Bacteroidaceae* and overall microbiota metabolic potential, while increasing levels of proinflammatory *Enterobacteriaceae* and lipopolysaccharide in bacterial cultures, reflecting shifts that occur *in vivo* in inflammation. Spent microbiota media induced elevated intracellular mucin levels and reduced intestinal monolayer integrity as reflected in transepithelial electrical resistance relative to fresh medium controls. However, multiplexed cytokine analysis revealed markedly different cytokine signatures from intestinal cultures exposed to spent medium with added oxidation products relative to spent control medium, while cytokine signatures of cultures exposed to fresh media were similar regardless of addition of host-derived cues. Further, the presence of immune cells in the intestinal model was required for this differentiation of cytokine signatures. This study indicates that simple *in vitro* immune-competent intestinal models can capture bacterial-mammalian cross-talk in response to host-derived oxidation products and supports utility of these systems for mechanistic studies of interactions between the gut microbiome and host in inflammation.

## Introduction

The human gastrointestinal tract harbors more than 10 trillion microbes^1^, collectively known as the gut microbiota. This complex community has recently been linked to numerous components of human health and disease, including conditions related to metabolic, immune, and brain health.^2^ Alterations to the microbiome due to infection, antibiotic treatment, a compromised immune system, or diet can result in microbial dysbiosis.^3^ Inflammatory conditions of the intestinal tract, such as inflammatory bowel disease (IBD), have been associated with an increased abundance of facultative anaerobes belonging to the class of Gammaproteobacteria, mostly *Enterobacteriaceae*, over obligate anaerobes, such as *Bacteroides* and short-chain fatty acids producing Firmicutes (e.g., clades IV and XIVa Clostridia).^4–8^ Oxidation products (e.g., nitrates, trimethylamine N-oxide (TMAO) and dimethyl sulfoxide) generated from host-derived antimicrobial radicals during intestinal inflammation can be utilized by Gram-negative bacteria of the *Enterobacteriaceae* family as terminal electron acceptors for anaerobic respiration, allowing for their rapid growth and successful niche establishment in the gut.^9–11^ The lipopolysaccharide (LPS) of the *Enterobacteriaceae* outer membrane is a highly reactogenic microbe-associated molecular pattern (MAMP) that promotes the secretion of proinflammatory cytokines, nitric oxide, and eicosanoids by monocytes, dendritic cells (DC), macrophages, and B cells.^12^ This cyclic process driven by the microbiome-host cross-talk can exacerbate intestinal inflammation.^13^

Animal models and advanced *in vitro* microphysiological systems have provided significant insight into host response to bacterial factors.^11,14^ The mucosal surface is the predominant site of interaction between the host and the gut microbiota and its derived metabolites, small molecule messengers, and MAMPs.^15,16^ The host epithelial layer produces a physical mucus barrier, which restricts bacterial penetration and molecule diffusion, and is composed of enterocytes, goblet cells, enteroendocrine cells, Paneth cells, and microfold cells. A simple *in vitro* cell culture model consisting of an intestinal absorptive epithelial cell line (Caco-2) and a mucus-producing goblet cell line (HT29-MTX) has been frequently used to represent the intestinal epithelium, for example to investigate the permeability of drugs,^17^ or to measure microbial adhesion.^18^ Recently, dendritic cells representing immune cells in the gut have been added to the Caco-2/HT29-MTX cell culture system to obtain a more representative intestinal model.^19,20^ Within the intestine, dendritic cells are recruited to injury sites and can extend dendrites into the lumen to capture resident bacteria,^21^ therefore contributing to the microbiota-host communication locally and systemically.

Herein, we investigated whether (1) *in vitro* cultures of microbiota populations would be modified in response to inflammatory oxidation products (exogenous nitrate and TMAO), and (2) a simple *in vitro* intestinal model is capable of capturing immunomodulatory effects of molecules released by microbiota populations in response to these cues. A simple gut simulator fermentation system (Lewis Gut Simulator – LEGS^22^) was utilized to cultivate human gut microbiota communities from a stool specimen in proinflammatory (exposure to sodium nitrate and TMAO) or control conditions. Filtered supernatant from these bacterial populations was then exposed to an immune-competent intestinal culture model of Caco-2/HT29-MTX/DC. Mucin production, monolayer integrity, and cytokine secretion were examined. Our results indicated modulation of microbiota structure and function *in vitro* in response to exposure to nitrate and TMAO mimicking the expansion of *Enterobacteriaceae* in conditions of intestinal inflammation. The shift in microbial population corresponded with reduced predicted metabolic capacity and increased endotoxin levels, and bacterial-derived molecules in the spent media resulted in a distinct cytokine profile in the intestinal model. This simple *in vitro* immune-competent gut model may reflect certain aspects of the exacerbated inflammatory response to pathobionts and oxidative stress in the gut, and thus could potentially be useful in screening therapeutic interventions.

## Results

**Figure 1.**
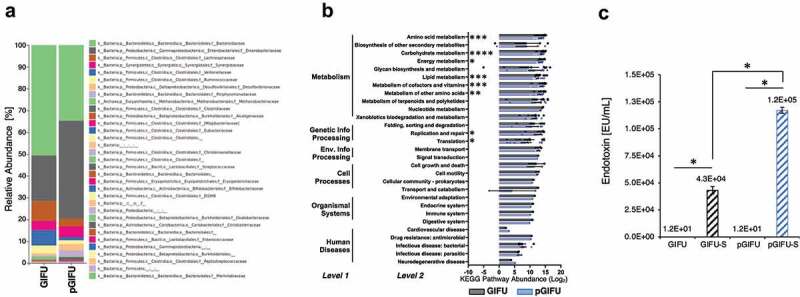
(a) Relative abundance at family level of taxonomy of GIFU and pGIFU microbiota. (b) Metagenome functional prediction by PICRUSt analysis at levels 1, 2 and 3 of KEGG pathways. Histogram bars indicate means of Log_2_(Level 3 pathway abundance) ± SEM clustered by level 2 (detailed in Supplement A), * *p* < .05; ** *p* < .01, *** *p* < .001, and **** *p* < .0001. (c) Endotoxin levels of different test media: fresh media GIFU and pGIFU, as well as spent media GIFU-S and pGIFU-S. * *p* < .05.

A human stool sample from a healthy donor was inoculated in a simplified human gut simulator (LEGS, Supplemental Fig 1S) in regular (GIFU) or proinflammatory (pGIFU; containing nitrate and TMAO) microbial media, and 1-week-old cultures were collected and analyzed by 16S rRNA V4 amplicon sequencing for microbial composition analysis. After a week of culture, the microbiota communities from the two different culture conditions comprised more than 20 bacterial families (Supplement A). The composition of the microbial population in pGIFU was severely altered as compared to the control GIFU culture (Figure 1a, Supplement A). For the most abundant families with a relative abundance greater than 10%, we observed a 2.2-fold increase in *Enterobacteriaceae* and a 1.5-fold decrease in *Bacteroidaceae* in pGIFU. Among bacteria present at an intermediate abundance of 1–10%, *Methanobacteriaceae* and several microbial families of the Firmicutes phylum (i.e. *Ruminococcaceae, Lachnospiraceae, Veillonellaceae*) were reduced in pGIFU. In contrast, *Clostridiaceae*, Proteobacteria members of the *Desulfovibrionaceae* family, and Bacteroidetes of the *Porphyromonadaceae* family were increased in pGIFU proinflammatory culture. Although possessing a similar species richness index in the alpha-diversity analysis (Supplemental Fig 2), the predicted overall genomic potential of the pGIFU microbial community was significantly reduced as compared to GIFU microbiota, especially in key metabolic functions, including metabolism of amino acids, carbohydrate, lipid, and vitamins, and general energy metabolism, as shown in the metagenomic KEGG pathway abundance analysis by PICRUSt (Figure 1b, Supplement A).

**Figure 2.**
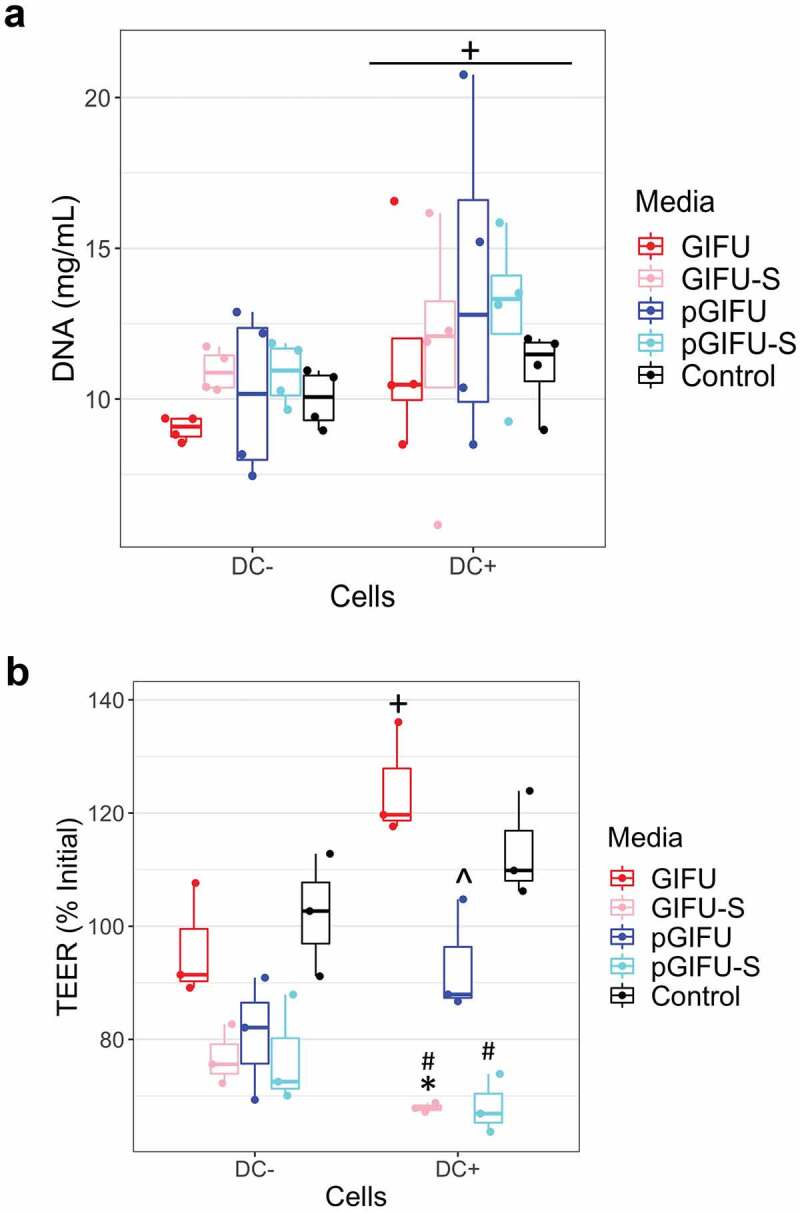
(a) DNA concentration of Caco2/HT29-MTX monolayers with and without dendritic cells (± DC) exposed to different test media. + *p* < .05 comparing all cultures -DC to +DC (main effect of cell model). (b) Transepithelial electrical resistance (TEER) indicating intestinal barrier function after 24-h exposure of monolayers to test media. * *p* < .05 comparing spent vs. fresh, ^ *p* < .05 comparing medium with proinflammatory factors vs. corresponding medium without proinflammatory factors, # *p* < .05 comparing to control serum-free intestinal cell culture media, with all comparisons within a given cell culture model, and ** *p* < .05 comparing -DC to +DC exposed to the same medium. All other comparisons are not significant. All measurements were collected from N = 3 biological replicates, and results are reported as an average ± standard deviation.

**Figure 3.**
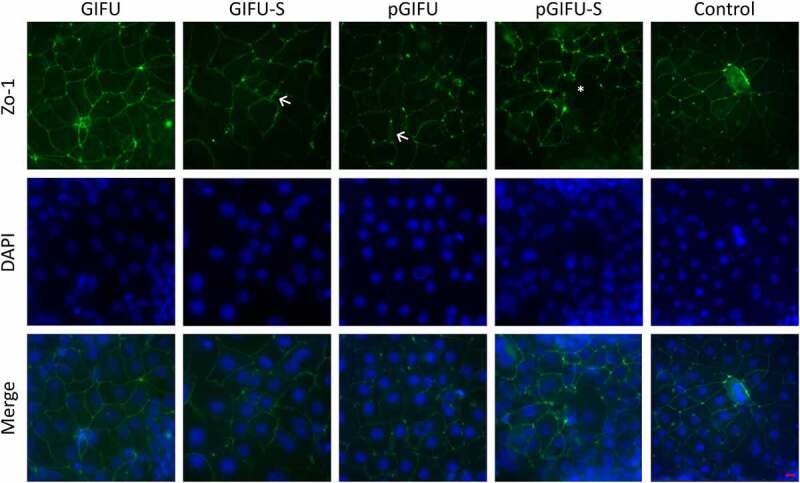
Cocultures with DC stained for tight junction (ZO-1, green) and DNA (DAPI, blue). (←) indicates areas of zig-zag ZO-1 staining pattern and (*) denotes low ZO-1 staining. Scale bar: 20 µm. A similar staining trend was observed for -DC cultures (data not shown).

In order to test the bulk of molecules released by the two microbiota communities, culture supernatants were filtered to remove bacterial cells and obtain spent media, GIFU-S and pGIFU-S. Pro-inflammatory pGIFU-S had significantly higher endotoxin levels compared with GIFU-S (Figure 1c). We then tested whether filtered spent media containing microbiota-derived molecules from the different gut simulators were able to differentially modulate homeostasis of intestinal culture models (Supplemental Fig 1S). Both “epithelial only” cultures (i.e. Caco-2/HT29-MTX coculture, -DC) and “immune-competent” cultures (i.e. Caco-2/HT29-MTX/DC coculture, +DC, with DC derived from a single donor) were tested. There was no significant difference in the number of epithelial cells after 24-h exposure to fresh vs. spent media, as shown by total DNA quantification (Figure 2a), indicating that the presence of microbial byproducts did not induce extensive cell death or proliferation. The incorporation of DC on the basolateral side of the Transwell® membrane did not significantly impact monolayer integrity in control cultures exposed to serum-free media (i.e. % change in Transepithelial electrical resistance (TEER) over 24 hof exposure to control serum-free media) when compared to cultures without DC. However, in +DC cultures, TEER was reduced with exposure to spent GIFU-S vs. fresh GIFU (*p* < .001), and also pGIFU-S vs. pGIFU, although not significantly (*p* = .057). In contrast, TEER of -DC cultures was not significantly impacted by exposure to spent relative to fresh media (Figure 2b). Exposure to spent media also significantly impacted TEER relative to exposure to control serum-free intestinal medium, again only in +DC cultures. Interestingly, proinflammatory nitrate and TMAO factors in fresh media significantly impacted TEER values, while the combination of residual nitrate, TMAO and their reduced derivatives from microbial anaerobic respiration in spent media did not, as seen in comparisons of +DC cultures exposed to GIFU vs. pGIFU and GIFU-S vs. pGIFU-S media. To directly observe changes to the monolayer, we stained for cell tight junctions, which regulate epithelial paracellular permeability,^23^ and we measured production of mucin, a major constituent of the intestinal mucosal barrier.^24^ In monolayers exposed to control serum-free intestinal medium and GIFU, ZO-1 tight junction staining appeared as a continuous belt-like structure (Figure 3). Monolayers exposed to GIFU-S and pGIFU had a zig-zag ZO-1 staining pattern, while pGIFU-S-exposed monolayers had areas of low staining. Low levels and zig-zag patterning of ZO-1 staining have been associated with decreased levels of this tight junction protein as well as increased permeability.^25,26^ Thus, this result suggests spent microbial media and supplementation with nitrate/TMAO impacted monolayer integrity.

**Figure 4.**
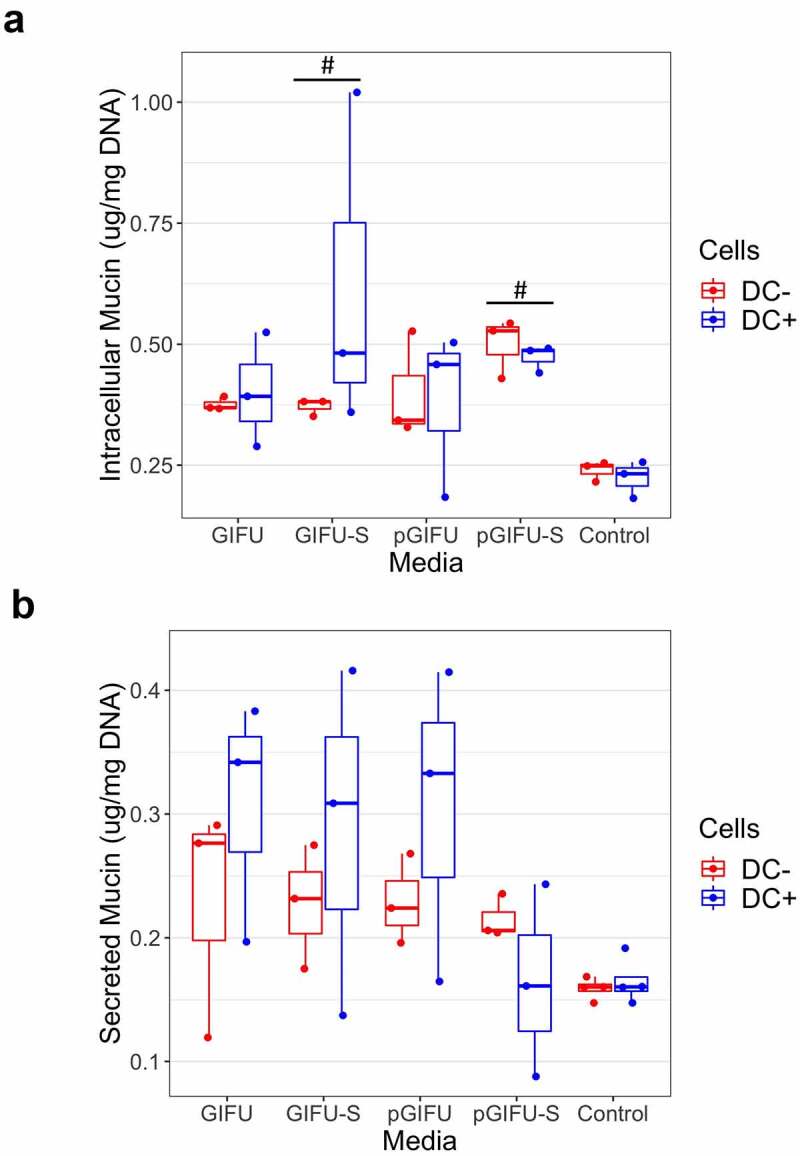
(a) Intracellular and (b) secreted mucin concentration from Caco2/HT29-MTX monolayers with and without dendritic cells (± DC) after exposure to test media. # *p* < .05 comparing to control serum-free intestinal cell culture media. All measurements were collected from N = 3 biological replicates and results are reported as an average ± standard deviation.

Exposure to spent microbial media affected the amount of intracellular mucin. We observed a statistically significant increase in intracellular mucin in monolayers cultured with or without DCs and exposed to spent media, i.e. GIFU-S and pGIFU-S vs. controls (Figure 4a). Levels of secreted mucin had greater variability (Figure 4b).

**Figure 5.**
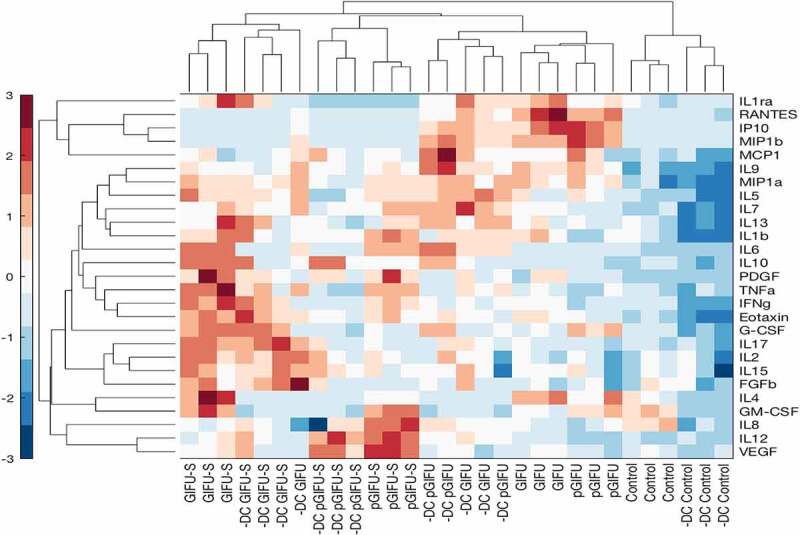
Hierarchical clustering of cytokine z-scores in different experimental groups. The cytokine levels are normalized to DNA concentration, mean-centered, and divided by standard deviation to generate z scores. Color bar indicates the relative cytokine amount, where red and blue correspond to high and low concentrations, respectively.

To assess whether the bacterial products in spent media, and, in particular, bacterial products in media containing proinflammatory factors, resulted in a measurable and specific immune response, apically secreted levels of 27 cytokines were quantified after exposure of intestinal monolayers to test media. Pairwise hierarchical clustering was performed to explore the similarity of cytokine response among samples (Figure 5). At the level of the third clave from the top, samples were grouped per medium into four clusters: control, pGIFU + GIFU, pGIFU-S, and GIFU-S (with only one GIFU outlier), and within each of these medium-clusters, samples were grouped by intestinal cellular model (± DC). This suggests that the medium, and specifically bacterial factors as well as the combination of residual nitrate, TMAO and their reduced derivatives from microbial anaerobic respiration in spent media were main driving forces of the cytokine response, which however differed for a given medium depending on the cellular model. Indeed, for most of the cytokines analyzed, the secreted levels were similar in overall magnitude between epithelial-only and immune-competent models, with the exception of IL4 and GM-CSF, which were markedly higher in cultures +DC for most media groups (Supplemental Fig 3). However, responses to bacterial factors in spent media relative to fresh media were generally more distinct in immune-competent models.

**Figure 6.**
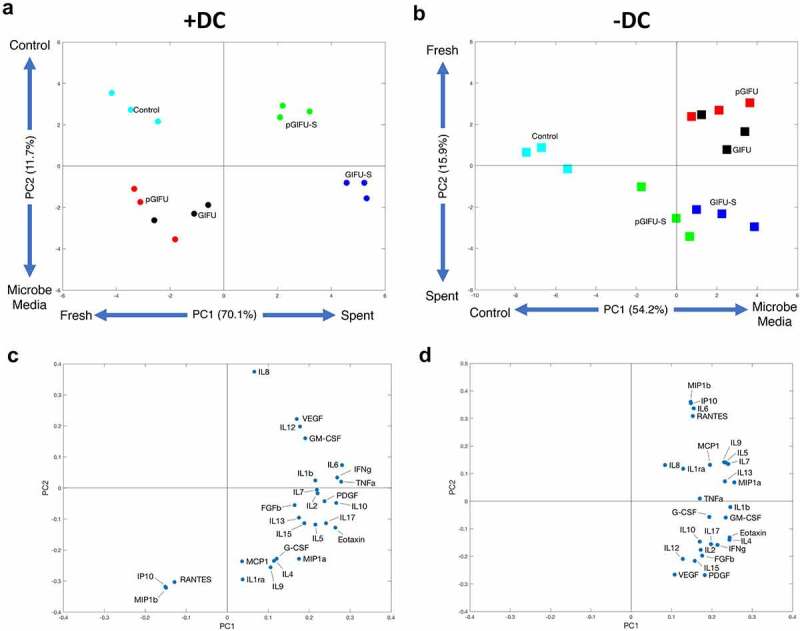
(a, b) Scores and (c, d) Loadings plots for a two-component PCA model constructed using measured cytokines in cultures (a, c) +DC and (b, d) – DC. The percentages noted reflect the percentage of variance in the data set captured in the latent variable.

Unsupervised PCA analysis revealed that two orthogonal linear combinations of cytokine levels, PC1 and PC2, captured 81.8% of the covariance in the dataset for cultures +DC (Figure 6a) and 70.1% for cultures -DC (Figure 6b). In cultures -DC, PC1 accounted for 54.2% of the variability, discriminating serum-free intestinal vs. microbial media, while PC2 accounted for 15.9% of the variability, discriminating fresh vs. spent bacterial media. In cultures +DC, PC1 accounted for 70.1% of the variability, discriminating fresh vs. spent media, while PC2 accounted for 11.7% of the variability, discriminating different types of fresh medium (serum-free intestinal control vs. GIFU-based media). Interestingly, a distinct response to spent bacterial media with and without the proinflammatory cues (pGIFU-s vs. GIFU-S), but not corresponding fresh media (pGIFU vs. GIFU), was reflected in PC1 and PC2 for immune-competent cultures, while cytokine profiles of cultures -DC did not reflect a distinct response to shifted bacterial factors resulting from exposure to proinflammatory cues (pGIFU-S vs GIFU-S).

The PCA loadings plot depicts the relative contribution of each cytokine to PC1 and PC2 (Figure 6c, D). In epithelial-only cultures, loadings on PC1 were positive for all cytokines, reflecting generally elevated levels in fresh and spent GIFU-based media relative to serum-free intestinal medium control. In immune-competent cultures, loadings on PC1 were positive for most cytokines, reflecting overall increase in inflammatory cytokine levels in cultures exposed to spent media relative to fresh media. However, exposure to fresh nutrient-rich GIFU media but not media that was spent by bacteria or serum-free intestinal medium control markedly elevated a small subset of immune mediators (IP10, MIP1b, and RANTES) as reflected in their negative and positive loadings on PC1 and PC2 in immune-competent and epithelial-only cultures, respectively (Figure 5, Supplemental Fig 3, Figure 6C, D), suggesting a potential response to nutritional cues.

Many measured cytokines showed a pattern of elevation in response to spent bacterial media relative to fresh medium in immune-competent cultures, as reflected in positive loading on PC1 (Supplemental Fig 3, Figure 6C), but did not show a differential response to proinflammatory cues (i.e. pGIFU-S vs. GIFU-S). IL6 response was most distinctly impacted by bacterial factors in spent media in immune-competent cultures, while levels in epithelial-only cultures were higher when exposed to fresh nutrient-rich bacterial media than in spent media and control, suggesting a potential differential response to nutritional or bacterial factors depending on the presence of immune cells in the model. GM-CSF levels were markedly higher in +DC relative to -DC cultures in most media groups, and significantly increased upon exposure of +DC cultures to spent media over fresh media. PDGF was also generally elevated in response to bacterial factors. Other cytokines reflected a clear response to bacterial factors in particular in immune-competent cultures, but at lower measured apical levels (< 10 pg/ml, Supplemental Fig 3), such as TNFα and IFNγ. Still other cytokines were at low apical levels and showed a more moderate response to bacterial factors, including IL1β, Eotaxin, and IL7.

A cytokine subset (i.e. IL8, IL12, and VEGF) showed a pattern of elevation in response to bacterial factors in pro-inflammatory spent GIFU medium relative to regular spent GIFU medium in cultures +DC, as reflected in loadings on PC2 (Figure 6c). Analysis of individual cytokine response did not reflect, however, a significant elevation in IL8 in response to pGIFU-S vs. GIFU-S, or a difference in IL12 or VEGF levels in cultures +DC vs. -DC. In contrast, some cytokines significant in maintenance of intestinal homeostasis (IL10, IL4) were notably more elevated in cultures +DC exposed to bacterial factors resulting from regular medium (GIFU-S) than from proinflammatory medium (pGIFU-S). IL1ra, G-CSF, IL17A, and FGFb levels showed a similar pattern, evident in both immune-competent and epithelial-only cultures.

## Discussion

A simplified gut simulator (LEGS) simulated shifts in the human gut microbiome when exposed to host-derived inflammatory cues. The increase in *Enterobacteriaceae* in pGIFU cultures supplemented with nitrate and TMAO (Figure 1a) mimics the *Enterobacteriaceae* bloom observed in the inflamed gut previously studied in different *in vivo* animal models of colitis,^9,10^ as well as in patients with ulcerative colitis and Crohn’s disease.^27^ The decrease in several families in the Firmicutes phylum and the increase in Proteobacteria in pGIFU cultures reflect a microbial signature reported in IBD patients^6^ and gnotobiotic mice with active colitis relative to those in remission.^28^ Specifically, Proteobacteria (*Enterobacteriaceae* and *Desulfovibri*o) were associated with colitis, and Actinobacteria, Firmicutes and Bacteroidetes with anti-inflammatory remission conditions.^28^ The alteration in microbial composition was accompanied by a shift in immunomodulatory and functional potentials. We observed an increase in proinflammatory LPS endotoxin via Limulus Amebocyte Lysate (LAL) assay. While Gram-negative *Enterobacteriaceae* generally possess a highly acylated and reactogenic form of LPS,^12^ *Bacteroides*, the predominant Gram-negative genus of *Bacteroidaceae* in the LEGS system (Supplement A), possess an LPS form with lower biological activity and reduced reactogenicity, so that abundance of *Bacteroides* has been negatively correlated with the LPS levels measured by the LAL test in fecal samples.^29,30^ Similarly in our model, increased measured endotoxin levels in pGIFU cultures correlated with an increased abundance in *Enterobacteriaceae* over *Bacteroidaceae*. This was accompanied by changes in the predicted microbial metabolic potential, with pGIFU microbiota being associated with a reduced capacity for energy harvest, similar to previous reports of studies in active *in vivo* colitis models.^28^ It is noted that the two microbial populations in GIFU and pGIFU media were derived from a single stool specimen, and taxonomic structure of the gut microbiota and its associated functional potential are individual dependent, and thus might vary if sampled across a larger donor population.

Microbial molecules and metabolites have been shown to influence cell proliferation, intestinal permeability and immune signaling both *in vitro* and *in vivo*.^31,32^ After 24-hr exposure to either fresh or spent microbial media, there was no impact on epithelial cell number (Figure 2a), which is consistent with previous work that showed that 24-hr exposure to 10 µg/ml LPS did not significantly affect cell number of Caco-2 cultures.^33^ However, TEER was significantly impacted by exposure to spent media only in immune-competent cultures, reflecting the important role immune cells play in sensing bacterial molecules. The decrease in TEER may be attributed to the increase in endotoxin levels in spent media compared to fresh media (Figure 2b), and may reflect a partial disruption of tight junctions, supported by imaging of ZO-1 (Figure 3), which has previously been implicated in the development of leaky gut in intestinal diseases.^23^

Within the gastrointestinal tract, extracellular mucin binds microbes preventing their penetration into the epithelium.^34^ Mucin synthesis and secretion can be activated by microbial products, toxins, and cytokines. For example, LPS from *Helicobacter pylori* and *Escherichia coli* alter mucin gene expression and secretion in human goblet cell lines and in rodent models.^35^ Although the fresh media do not contain any microbial products or toxins, the rich nutritional content of the bacterial medium may have impacted mucin production, although no significant effects were observed (Figure 4). The addition of dietary fibers (i.e. 0.5 g/L soluble starch in GIFU media) has been previously shown to increase goblet cell volume in the small intestine.^36^ Dietary fibers can also be digested by members of the microbiota resulting in the synthesis of short-chain fatty acids, which have also been shown to increase MUC2 mRNA levels in goblet-like LS174T cells,^37^ and may have contributed to the observed increase in intracellular mucin levels in cultures exposed to spent media.

The analysis of the intestinal model cytokine response suggested activation of signaling pathways that are relevant to host-microbial interactions.^38^ Detected cytokine levels in epithelial cultures -DC reflect the fact that not only immune cells, but also the intestinal epithelium is in itself an important producer of cytokines.^39^ Reports of production of cytokines by the epithelium, and significance of cytokines specifically to intestinal inflammation, are noted in the discussion below. The overall similar absolute cytokine levels in epithelial-only and immune-competent models (Supplemental Fig 3) likely is due in part to the measurement of apical cytokines, mostly influenced by epithelia present at the apical interface. Similarly, coculture of primary small intestinal epithelium with macrophages was not reported to change apical levels of IL8, IFNγ or IL6, while basolateral levels increased markedly with the presence of macrophages.^40^ Indeed, the levels of apical factors analyzed in this study likely better reflect values of intestinal lumen contents, but might vary significantly from basolateral secretion levels for some cytokines.^40–42^ The incorporation of immune cells did distinctly alter cytokine profiles after exposure to fresh or spent microbial media (Figure 5, Figure 6, Supplemental Fig 3). It is possible that some reported cytokine levels reflect both basolateral secretions from DC and apical factors modulated by the epithelial-immune crosstalk, given the ability of DC to infiltrate the epithelium,^21^ and the increased permeability, reflected in TEER, of cultures exposed to bacterial factors. In interpreting the response of DC-containing cultures, it is important to recall that DC were derived from a unique blood donation, and response may likely vary with different donors.

Most of the cytokines assessed in the multiplex panel showed a general pattern of elevation upon exposure to bacterial factors in spent as compared to fresh media, especially in immune-competent cultures (i.e. significant differences between both GIFU-S and pGIFU-S with respective fresh media were observed for IL6, GM-CSF, TNFα, and INFγ in +DC cultures) (Figure 5, Figure 6c‒D, and Supplemental Fig 3). Notable exceptions include elevation in fresh vs. spent media of IP10, MIP1β, and RANTES in both +DC and -DC cultures, and IL6 and IL7 in -DC cultures (Figure 5, Figure 6c‒D, and Supplemental Fig 3). This may indicate a response to nutritional cues which are depleted in spent media due to microbial metabolism.

Cytokines elevated in spent vs. fresh media have known roles in response to bacterial factors and intestinal inflammation. IL6, a pleiotropic cytokine central in regulating inflammatory responses, is released by both immune (e.g. monocytes and macrophages), and epithelial cells,^43^ and is important in attracting inflammatory cells and activating T and B lymphocytes. Together with other inflammatory cytokines such as TNFα and IFNγ, elevation of IL6 has been demonstrated to negatively affect the intestinal epithelial barrier function,^39^ in agreement with what we observed in immune-competent cultures exposed to spent media (Figure 2b). TNFα, a central immune cytokine produced by DC, stimulates T-cells to produce IFNγ.^44^ TNFαααproduction by intestinal epithelial cells has been implicated in intestinal inflammation, with most significant secretion likely occurring basolaterally to impact underlying mesenchymal cells.^45^ IFNγ is produced mainly by T lymphocytes and NK cells, but production by epithelial cells and DCs has been reported.^46^ GM-CSF has been demonstrated to be produced by colon epithelium and plays an important role in impacting intestinal epithelial proliferation in response to injury^47^ as well as recruitment of DC in response to pathogen invasion.^48^

Factors elevated in response to fresh vs. spent media, potentially due to nutritional cues present in fresh media, also are important in intestinal immune function. IP10, MIP1b, and RANTES are produced by intestinal epithelia as well as immune cells and have been implicated in inflammatory, metabolic or diet-related conditions.^49–52^ The shift of IL6 and IL7 from being elevated in fresh media in epithelial-only cultures to being elevated in spent media in +DC cultures reflects the significance of immune cells in the mucosal model’s response to bacterial factors. Intestinal epithelial cells also produce IL7,^53^ which can impact IL4 production by immune cells.

The differential response to spent vs. fresh pGIFU and GIFU media observed for levels of IL1ra and G-CSF could signify suppression of these cytokines by factors present in the proinflammatory bacterial cultures. IL1ra has been demonstrated to be produced at elevated levels at the apical surface of cervical epithelium,^54^ and at biologically relevant (with respect to modulating IL1β) levels by oral mucosal epithelium.^55^ IL1ra plays the important role of inhibiting the effect of pro-inflammatory IL1β by binding to its receptor, and its modulation by microbial factors has been associated with reduced intestinal inflammation and tissue damage.^56,57^ G-CSF plays a role in maintenance of homeostasis^58^ and is important in promoting survival of recruited polymorphonuclear cells in the intestine. It has been used as an efficacious therapy in some patients with Crohn’s disease.^59^ G-CSF was shown to be produced by airway epithelium in response to bacterial stimulation.^60^

The incorporation of immune cells in our intestinal model was not only required for bacterial stimuli to be the major discriminating factor in cytokine signature, but was also key for inducing a differential cytokine signature to pro-inflammatory microbiota versus control microbiota, as reflected in separation of pGIFU-S and GIFU-S on the principal component scores plot (Figure 6, Supplemental Fig 3). In immune-competent cultures, the change in bacterial factors in response to pro-inflammatory cues (pGIFU-S vs GIFU-S) was reflected in positive loadings on principal components 1 and 2 on the scores plot for IL8, IL12, and VEGF (Figure 6a). IL8 is produced by multiple cell types including monocytes and epithelial cells and regulates host response to microbial factors by acting as a chemoattractant for neutrophils. It has been demonstrated that bacteria differentially modulate production of IL8 by epithelial cells.^61^ Polarized (apical) secretion of IL8 by intestinal epithelium has been demonstrated to be stimulated by ligand binding to Toll-like receptor 2 (TLR2) and TLR5,^42,62^ which are major receptors for MAMPs including LPS, lipoproteins and flagellin.^63^ IL12 has been reported to be produced mainly by monocytes in the intestine, but airway epithelium has been demonstrated to produce IL12 in inflammation,^64^ and elevation of this Th1-inducing factor has been observed in intestinal inflammatory conditions in humans and animal models.^65^ VEGF is a key mediator of angiogenesis, a process highly integrated with inflammation, is produced by intestinal epithelial cells in response to microbial stimuli, and impacts epithelial as well as vascular biology.^66,67^

In contrast to cytokines elevated in response to pro-inflammatory bacterial factors (pGIFU-S vs. pGIFU), there were several cytokines that showed the opposite trend. Average induction of cytokines by bacterial factors in proinflammatory media (i.e. level in pGIFU-S/level in pGIFU) was relatively lower than induction in regular bacterial media (i.e. level in GIFU-S/level in GIFU) for IL10, IL4, IL17A, and FGFb (Figure 5, Figure 6c, Supplemental Fig 3), as well as IL1ra and G-CSF, which were discussed above in light of decrease in pGIFU-S vs. pGIFU. IL10 has been long recognized as a key mediator of intestinal immune regulation by the microbiota.^68^ IL4 has anti-inflammatory and inflammatory actions depending on context, and is able to promote release of IL10, as well as TGFβ regulatory cytokines.^69^ IL4 is the most potent polarizing cytokine for Th2 differentiation, primarily produced by Th2 cells in the gut and by a subset of monocyte-derived inflammatory DC.^70^ Thus, elevated IL4 in DC-containing cultures could be a result of production by DC, or, as the DC were derived from human leukocytes and likely do not represent a pure population, by other leukocyte populations. IL4 expression in Caco-2 and HT29 cell lines has also been previously reported.^71^ IL4 is a potent inhibitor of IL8^72^, which may in part explain elevated levels of IL8 in immune-competent gut models exposed to pGIFU-S relative to GIFU-S. IL17A, which is produced by both immune and nonimmune cells, was relatively low in expression level, but is central in governing a protective immune response at mucosal sites and regulating interactions with the microbiota. FGFb has been demonstrated to be secreted at significant levels, which are elevated in inflammatory bowel disease, at the apical intestinal mucosal surface.^73^

Overall, several cytokines suppressed by pGIFU-S relative to GIFU-S, including IL1ra and G-CSF discussed above, have known roles in inhibiting inflammatory cytokines and/or maintenance of homeostasis. Combined with elevation of inflammatory cytokines (IL8, IL12, VEGF), the specific cytokine signature observed upon exposure to pGIFU-S media suggest that molecules produced by the microbiota in response to inflammatory conditions (nitrate and TMAO), reflecting altered microbial composition and associated elevated endotoxin levels, produce a dominant and distinct pro-inflammatory response which may disrupt the immunological equilibrium. On the contrary, increased levels of some cytokines, such as IL10, in models exposed to regular GIFU-S may reflect cellular measure to counteract excessive monolayer-damaging inflammation and could be induced by regulatory microbial molecules or other medium components to restore homeostasis.^74^

It is noted that cytokines actively trigger signaling in the 5–100 pM range. Many of the values reported are below or at the lower end of this range. Other recent reports from primary intestinal cells (as opposed to intestinal cell lines employed here) showed higher levels of some cytokines,^40,62^ including apical IL8 and MCP1, being more than an order of magnitude greater than what was measured here. These disparities might reflect differences between primary and cell line cultures, and in culture conditions (culture area, media type and volume, time of sampling). However, levels of IL8^42,74^ and MCP1^75^ previously measured in Caco-2 cultures, as well as of VEGF in T84 cell line^66^ were comparable to those measured here. IFNγ and IL6 amounts secreted in apical media of primary colonic epithelium were similar to those reported here for Caco-2/HT29 cultures.^40^ Also, low levels of some inflammatory cytokines may reflect their primary production *in vivo* by cells not present in the intestinal cultures employed in these studies. Finally, our model can be considered to approximate the function of the colon, as both Caco-2 and HT29 are derived from colonic tissue, and the microbial consortia were derived from stool. However, primary cultures derived from a specific part of intestine (e.g. jejunum or colon) could be used to more precisely model region-specific intestinal functions.

The varied response of cytokines observed in this study reveals an intricate modulation of host-microbiota cross-talk. It is noted that only multiplex cytokine analysis capturing a broad range of secreted factors, paired with PCA and hierarchical analyses, clearly differentiated the variable response to bacterial factors reflecting a shift to an altered bacterial population induced by proinflammatory factors, while all other analyses described above (i.e. monolayer integrity, ZO-1 staining, mucin production) reflected a marked response only to spent vs fresh media. Further, if select factors had been analyzed (e.g. TNFα and IL6) as opposed to a broad array of cytokines and chemokines, the differential response to the proinflammatory culture may not have been evident.

## Conclusion

Microbial communities prepared in a simple gut simulator (LEGS) can be utilized in combination with human intestinal cultures to study immune-mediated cross-talk between the microbiota and the host. The supplementation of host-derived metabolites produced during inflammation (nitrate and TMAO) resulted in a bloom of proinflammatory *Enterobacteriaceae* which was accompanied by elevated endotoxin levels and reduced metabolic potential, compared to microbial communities cultured in regular GIFU media. Caco-2/HT29-MTX/DC monolayer integrity, as well as the production of mucin were altered upon exposure to spent media from bacterial cultures, but these metrics did not differentiate response to regular GIFU-S relative to inflammatory pGIFU-S media. Multiplex cytokine analysis, however, revealed distinct cytokine signatures differentiating the response to fresh versus spent media, and importantly the response to spent pro-inflammatory vs. control bacterial GIFU media. The incorporation of DC in the intestinal culture model was required for differential response to the proinflammatory (pGIFU-S) vs control spent bacterial (GIFU-S) medium as reflected in PCA. Finally, PCA revealed a subset of elevated cytokines (i.e. upregulated IL8, IL12, VEGF; and downregulated IL1ra) reflecting an exacerbated inflammatory response to metabolites and molecules generated by the proinflammatory microbiota as compared to the control microbial community. These results highlight the utility of a simple immune-competent *in vitro* intestinal models paired with multiplex cytokine analysis as a useful tool in recapitulating and providing insight into the microbial and immune signatures observed in intestinal inflammation. Such models can be explored for in-depth analysis of microbiome-gut-immune cross-talk, as well as to screen potential therapeutic interventions. Further investigation of variability of response with host and microbial components derived from a broader pool of donors will be highly important in advancing application of this model.

## Materials and methods

### Lewis gut simulator (LEGS) and microbiota spent media

GIFU anaerobic medium (HiMedia®) composed of 10 g/L peptone, 3 g/L soya peptone, 10 g/L protease peptone, 13.5 g/L digested serum, 5 g/L yeast extract, 2.2 g/L meat extract, 1.2 g/L liver extract, 3 g/L dextrose, 2.5 g/L potassium dihydrogen phosphate, 3 g/L sodium chloride, 5 g/L soluble starch, 0.3 g/L L-cysteine hydrochloride, and 0.3 g/L sodium thioglycolate, was diluted 1:10 and maintained at constant pH 7 for the whole course of the experiment by the addition of 50 mM 3-(N-morpholino)propanesulfonic acid (MOPS, ThermoFisher Scientific) buffer. Proinflammatory GIFU (pGIFU) was composed of 1:10 diluted GIFU anaerobic media supplemented with 4 mM sodium nitrate (Sigma Aldrich) and 4 mM TMAO (Sigma Aldrich).

The collection of human stool samples from healthy volunteers was approved by Northeastern University IRB# 08–11-16. Written consent was obtained from the donor. A LEGS gut simulator was used to culture human gut communities from a single healthy human donor in either GIFU or pGIFU media under anaerobic conditions (vinyl anaerobic chamber, Coy Laboratory Products, Inc.). The system was built and operated as previously described,^22^ with some modifications (Supplemental Fig 1S). For each media type, a bottle of sterile deoxygenated medium was attached to a sequential two-chamber chemostat. Sterile silicone tubing (3/32” ID × 5/32” OD) connected the media vessel to the first chamber, which was connected to the second chamber via additional 3/32” ID × 5/32” OD tubing and peristaltic tubing (size 0.89 mm). 1 ml aliquots of the donor stool sample diluted 10^5^ in deoxygenated medium were inoculated into both chambers on the first day of the experiment, and then media was pumped into the first chamber so that the entire volume (150 mL) was replaced every 24 hrs. The system is designed such that components of the fresh medium are utilized by the microbial community, and the breakdown products of metabolism are then transitioned to the second chamber (akin to what happens in the human GI tract) at the same rate fresh media is pumped into the first chamber. For the present experiment, a single LEGS system was prepared with each medium (GIFU and pGIFU), and after one week of culture, the microbial community from the second chamber was collected for V4 16S rRNA amplicon sequencing, pelleted by centrifugation, and spent medium was 0.22 µm filtered to remove live and dead bacteria, and stored at −80°C prior to use in *in vitro* culture models. These supernatant samples will be referred to as GIFU-S and pGIFU-S for spent regular and proinflammatory GIFU media, respectively. Viability of the LEGS communities at day 7 was confirmed by CFU plating on solid media, and no significant difference in CFU counts was observed between the two culture conditions (data not shown).

### 16S rRNA amplicon sequencing and microbiota dataset analysis

DNA extraction and sequencing were performed by Mr. DNA (Shallowater, TX, USA) with a Roche 454 FLX. Amplification of the V4 variable region was performed using PCR primers 515 F/806 R in a 30 cycle PCR with the HotStarTaq Plus Master Mix Kit (Qiagen, USA). The following conditions were used: 94°C for 3 minutes and 30 cycles of 94°C for 30 seconds, 53°C for 40 seconds and 72°C for 1 minute, followed by a final elongation step at 72°C for 5 minutes. Data were demultiplexed and quality filtered in and analyzed using Quantitative Insights into Microbial Ecology (QIIME™).^76^ Operational taxonomic unit (OTU) and feature tables were generated, respectively, in QIIME1 using a closed-reference protocol based on the Greengenes database^77^ and in QIIME2^78^ using the vsearch dereplicate-sequences command. Taxonomy was assigned to the feature table using the Greengenes 13–8 classifier, and the family and genus level relative abundance is reported. The functional contents of the metagenome were predicted using PICRUSt^79^ using default parameters based on the closed-reference OTU table. Identified gene families (KEGG Orthology groups) were grouped into metabolic pathways based on the BRITE hierarchy, and relative abundance of clustered Level 3 pathways was compared (Supplement A).

### Quantification of endotoxin levels in microbiota spent media (LAL assay)

Endotoxin concentration in fresh and spent media was quantified using Pierce Limulus Amebocyte Lysate Chromogenic Endotoxin Quantification Kit (ThermoFisher Scientific). Briefly, a microplate was equilibrated on a heating block for 10 min at 37°C. 50 μl of each test medium (GIFU, pGIFU, GIFU-S, or pGIFU-S) or standard was added to a separate well, followed by addition of 50 μl of LAL solution and incubation for 10 min at 37°C. The chromogenic substrate solution (100 μl) was added to each well and incubated for 6 min at 37°C. Finally, 50 μl of the stop reagent (25% acetic acid) was added, and the absorbance was measured at 405 nm using a BioTek Powerwave XS spectrophotometer. The standard curve ranged from 0 to 1.0 endotoxin units (EU)/ml and was used to calculate the concentration of the samples that were run in triplicate.

### In vitro cell culture model

Human intestinal carcinoma epithelial (Caco-2 clone C2BBe1, ATCC) and mucus-producing (HT29-MTX-E12, Sigma Aldrich) cells were cultured in Advanced Dulbecco’s Modified Eagle’s Medium (DMEM, ThermoFisher Scientific) supplemented with 10% heat-inactivated fetal bovine serum (Atlanta Biologics), 1× GlutaMax™ (ThermoFisher Scientific), and 1x Penicillin/Streptomycin (P/S, ThermoFisher Scientific). Caco-2 (passage 55–65) and HT29-MTX (passage 35–45) cells were seeded in 9:1 ratio on 24 well Transwell® inserts (Falcon™) at a density of 10^5^ cells/cm.^2^ Briefly, the apical and basal sides of the Transwell® membrane were coated with 50 μg/ml collagen type I from rat tail (Corning®) in phosphate buffered saline (PBS, ThermoFisher Scientific) for 2 h at room temperature. Caco-2 and HT29-MTX were harvested from culture flasks using 0.25% Trypsin-EDTA (ThermoFisher Scientific) to obtain single cells for seeding onto the apical side of the Transwell® membrane. Culture medium was replaced every 2 days (apical: 300 µl, basolateral: 700 µl). One week post-seeding, the medium was changed to serum-free intestinal medium composed of Advanced DMEM, 1× GlutaMax^TM^, 1× P/S, 1× Insulin-Transferrin-Sodium Selenite (Roche Diagnostics).

DC derived from human blood monocytes were incorporated as the immune component of the coculture model^19,20^ (Supplemental Fig 1S). Briefly, peripheral blood mononuclear cells were isolated from Human Peripheral Blood Leuko Pak (STEMCELL^TM^ Technologies) using EasySep^TM^ Human Monocyte Enrichment Kit (STEMCELL^TM^ Technologies). Monocytes were differentiated into DC using Advanced RPMI medium (Gibco) supplemented with 1× GlutaMax^TM^, 1% P/S, 50 ng/ml granulocyte/macrophage colony-stimulating factor (GM-CSF, Biolegend®), 35 ng/ml interleukin 4 (Biolegend®), and 10 nM retinoic acid (Sigma Aldrich). After 7 days of differentiation, DC were harvested using accutase™ (STEMCELL^TM^ Technologies) and seeded on the basolateral side of the Transwell® membrane used to culture Caco-2/HT29-MTX for 2 weeks as described above, at a 1:10 ratio of DC:epithelial cells. Briefly, apical medium was removed, and the Transwell® was gently inverted. 75 µl DC suspension was added to the membrane and incubated for 30 min at 37°C and 5% CO_2_, after which the Transwell® was gently placed back into the 24 well culture plate. Caco-2/HT29-MTX cultures in Transwell® inserts with and without DC were maintained for an additional week in serum-free media. Medium was changed every other day.

### Exposure of intestinal cells to microbiota spent media

Cells in the *in vitro* model were exposed to microbiota spent (GIFU-S or pGIFU-S) or fresh (GIFU or pGIFU) GIFU-based media, or serum-free intestinal cell culture media as control. Apical and basolateral chambers were washed with 500 μl of PBS prior to exposure to test media. PBS in the apical chamber was removed and replaced with 300 μl of test medium, PBS in the basolateral chamber was removed and replaced with 700 μl serum-free intestinal medium, and the cultures were incubated for 24 h at standard cell culture conditions (37°C and 5% CO_2_).

Transepithelial electrical resistance (TEER) measurements were made prior to exposure to test media and after 24-h exposure. An EndOhm-24SNAP chamber with an EVOM2 meter (World Precision Instruments) was used to measure TEER. Samples and media were kept warm at 37°C prior to measurement to minimize variability between TEER measurements due to temperature fluctuations. Percent change from the original TEER measurement was calculated. Prior to exposure to test media, measured TEER values ranged from 250–300 Ohms/cm.^2^

After 24-h exposure, apical and basolateral media samples were collected and stored at −80°C. Caco-2 and HT29-MTX cells were lysed to analyze intracellular mucin concentration. Briefly, apical and basolateral chambers were each washed with 500 μl PBS. After removing PBS from both chambers, cells were placed overnight at −80°C. The plate was then thawed on ice for 10 min and 500 μl lysis buffer (50 mM Tris(hydroxymethyl)aminomethane, 10% glycerol, 150 mM sodium chloride, 1% NP-40, all chemicals from Sigma Aldrich) was added to each apical compartment. The Transwell® membrane surface was then scraped with a pipet tip to remove all cells, and scraped material was collected in a microcentrifuge tube. Each sample was vortexed for 1 min and placed on ice for 30 min on a shaker. A mini bead beater mill (Cole-Parmer®) was used to homogenize the sample for 20 s. Samples were centrifuged at 10,000 g for 10 min at 4°C to remove cellular debris, and the supernatant was analyzed for mucin and DNA concentration.

### Mucin quantification

Secreted and intracellular mucin concentrations were quantified using the alcian blue assay.^80^ Briefly, 100 μl of sample was loaded into a 96 well plate and 33.4 μl of alcian blue (Sigma Aldrich) was added. The plate was equilibrated on a shaker for 2 h at room temperature and then centrifuged for 30 min at 1870 xg. The plate was washed twice with 100 μl of wash buffer (40% ethanol, 0.1 M sodium acetate, 25 mM magnesium chloride, at pH 5.8, all chemicals from Sigma Aldrich). The pellet was then resuspended in 100 μl of 10% sodium dodecyl sulfate (Sigma Aldrich). Sample absorbance was measured at 620 nm using a BioTek Powerwave XS spectrophotometer. A standard solution of 0–250 μg/ml mucin from bovine submaxillary glands (Sigma Aldrich) was used to quantify the concentration of unknown samples.

### DNA quantification

CyQUANT® cell proliferation assay kit (ThermoFisher Scientific) was used to measure DNA concentration. Briefly, the lysed cell supernatant was supplemented with 1 mM ethylenediaminetetraacetic acid (EDTA, Sigma Aldrich) to prevent DNA degradation. In a 96 well plate, 100 μl of standards, samples, or blank and 100 μl of CyQUANT® GR dye was added and incubated for 5 min at room temperature. The fluorescence of the samples (480/520 λ_ex_/λ_em_) was measured using EnSight^TM^ multimode plate reader (PerkinElmer, Inc.). Bacteriophage λ DNA was used to make a standard curve ranging from 0 to 1000 ng/ml.

### Caco-2/HT29-MTX monolayer staining for ZO-1

After exposure to test media, apical and basolateral chambers were washed with 500 μl PBS, fixed with 500 μl 4% paraformaldehyde (Sigma Aldrich) in PBS overnight at 4°C, washed with 1× PBS, and permeabilized with 0.4% Triton-X (Sigma Aldrich) in PBS for 30 min at room temperature. To minimize nonspecific interactions, apical and basolateral chambers were blocked with 2.5% goat serum (500 μl, Sigma Aldrich) in PBS for 30 min at room temperature. The tight junction protein zonula occluden (ZO-1) was stained with Alexa Fluor 488 conjugated ZO-1 monoclonal antibody (5 µg/ml, apical chamber only, ThermoFisher Scientific) overnight at 4°C. Cell nuclei were stained with 4’, 6-Diamidino-2-Phenylindole, Dihydrochloride (DAPI, 300 nM, apical chamber only, ThermoFisher Scientific) for 30 min at room temperature. Images of the stained monolayer were obtained using Zeiss Axio Observer Z1 with attached X-Cite® 120LED and Hamamatsu ORCA-Flash 4.0 Digital Camera.

### Multiplex cytokine assay

Cytokine levels were measured using Bio-Plex Pro^TM^ Human Cytokine 27-plex Assay (Bio-Rad Laboratories, Inc.). To minimize nonspecific binding of proteins to antibody-coupled magnetic beads, bovine serum albumin (BSA) was added to each sample to achieve a final concentration of 5 mg/ml. The protein standard was diluted in serum-free medium supplemented with BSA. The assay was run according to manufacturer protocol. Briefly, 50 μl beads were added to each well, and each well was then washed 2× with 100 μl Bio-Plex wash buffer using Bio-Plex Pro^TM^ Wash Station. Next, 50 μl of samples, standards, or blank was added to each well and incubated on a shaker at 850 rpm for 1 h at room temperature. The wells were washed 3× with 100 μl wash buffer, 1× detection antibody was added, and the plate was incubated on a shaker at 850 rpm for 1 h at room temperature. The wells were again washed 3× with 100 μl wash buffer, 50 μl Streptavidin, R-Phycoerythrin Conjugate (SA-PE) was added, and the plate was incubated on a shaker at 850 rpm for 10 min at room temperature. After a final 3× wash with 100 μl wash buffer, the beads were resuspended in 125 μl of assay buffer and incubated on a shaker at 850 rpm for 30 s. The plate was analyzed using a Bio-Plex^TM^ 200 System and Bio-Plex^TM^ Manager 6.1. The concentration of each cytokine was determined from the standard curve, which was generated by fitting a five-parameter logistic regression of mean fluorescence on known cytokine concentrations. Samples were diluted to ensure the measurements were within the linear dynamic range of the assay.

### Multivariate analysis

Cytokine level data were normalized by mean-centering and unit scaling variance prior to hierarchical clustering and principal component analysis (PCA). Hierarchical clustering was completed using the *clustergram* function in MATLAB® software (R2017B, Mathworks, Inc.), which clusters data using Euclidean distance metric and average linkage. Mean centering is the subtraction of the mean from each individual value. Unit variance scaling is dividing the mean-centered data by the standard deviation of the mean-centered data. PCA was completed using MATLAB® software with the *pca* function. In PCA, the linear combination of cytokines that explains the highest possible amount of variance in the data is iteratively identified, and then removed from the data, converting the original cytokine data into linearly uncorrelated variables, called principal components (PC).

### Statistical analysis

PICRUSt data in Figure 1b are presented as mean ± standard error of the mean (SEM), and the Wilcoxon matched-pairs signed rank test was used to assess significance with p < .05. The dependences of DNA concentration, TEER, mucin concentration, and cytokine concentrations on test media, dendritic cells, or the interaction of the two (Figures 2, 4, and Supplemental Fig 3), were analyzed by two-way ANOVA using version 4.1.0 of R.^81^ The significance of difference between individual levels of significant main or interaction effects was assessed post-hoc by Tukey’s Honest Significant Difference method. Complete results reported in Supplement B were generated using the R packages openxlsx (v. 4.2.4) ggplot2 (v. 3.3.5,^82^) and ggbeeswarm (v 0.6.0). Data in Figures 2 and 3 as well as Supplemental Fig 3 are presented as box plots generated in R.

## Supplementary Material

Supplemental Material
