# Supplementary material for: An in vitro intestinal model captures immunomodulatory properties of the microbiota in inflammation
Source: Gut Microbes. 2022 Mar 22;14(1):2039002. doi: 10.1080/19490976.2022.2039002 (PMC8942420; doi:10.1080/19490976.2022.2039002)
Supplement: Supplemental Material [file KGMI_A_2039002_SM4653.zip › supplementary/Suppl Fig 1.pptx]

## Slide 1
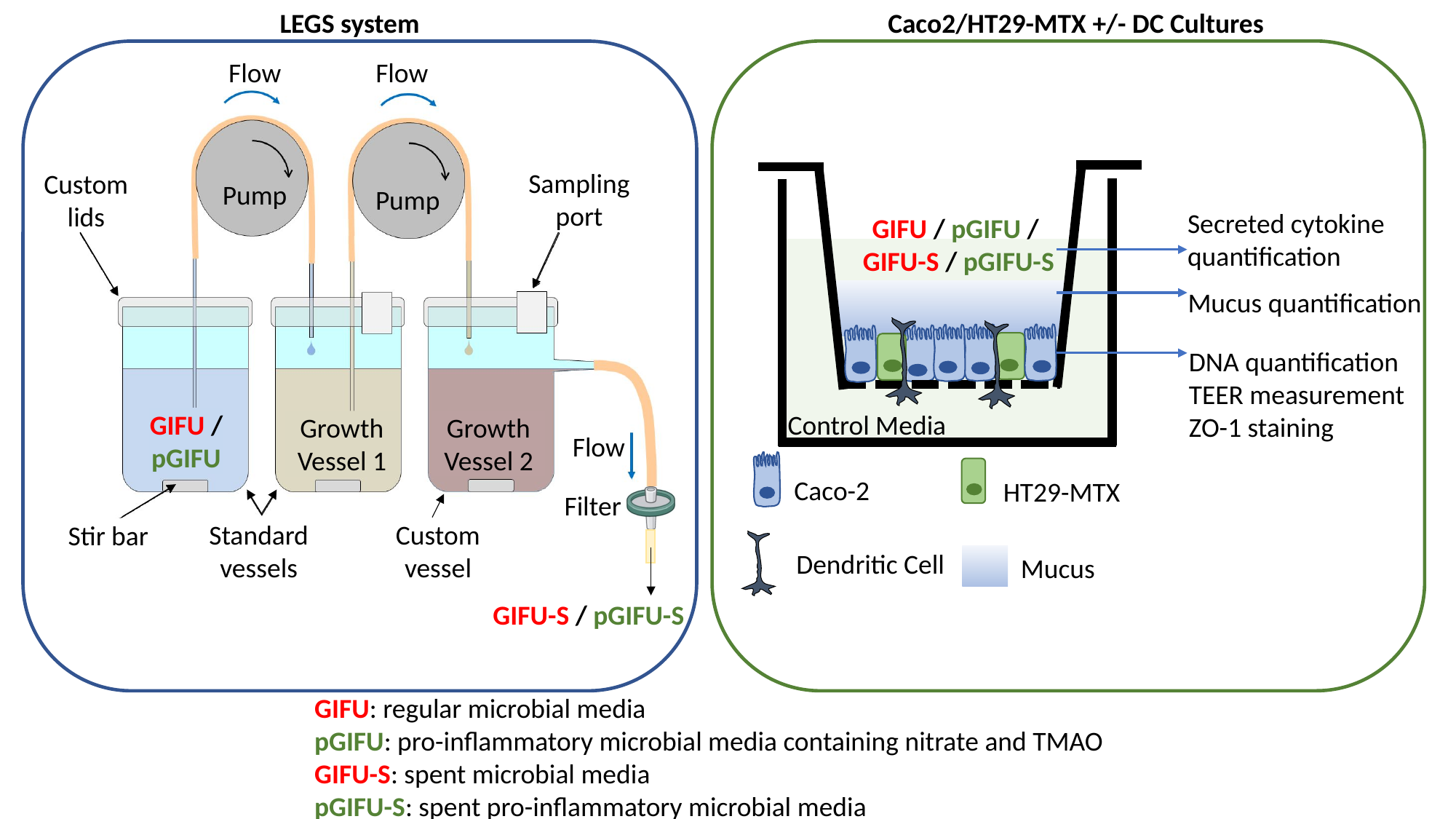

LEGS system
Caco2/HT29-MTX +/- DC Cultures
Flow
Flow
Sampling port
Custom lids
Pump
Pump
GIFU / pGIFU
Growth Vessel 1
Growth Vessel 2
Flow
Filter
Standard vessels
Custom vessel
Stir bar
GIFU-S / pGIFU-S
GIFU / pGIFU /
GIFU-S / pGIFU-S
Control Media
Secreted cytokine quantification
Mucus quantification
DNA quantification
TEER measurement
ZO-1 staining
Caco-2
HT29-MTX
Dendritic Cell
Mucus
GIFU: regular microbial media
pGIFU: pro-inflammatory microbial media containing nitrate and TMAO
GIFU-S: spent microbial media
pGIFU-S: spent pro-inflammatory microbial media
