# Supplementary material for: An in vitro intestinal model captures immunomodulatory properties of the microbiota in inflammation
Source: Gut Microbes. 2022 Mar 22;14(1):2039002. doi: 10.1080/19490976.2022.2039002 (PMC8942420; doi:10.1080/19490976.2022.2039002)
Supplement: Supplemental Material [file KGMI_A_2039002_SM4653.zip › supplementary/Suppl Fig 2.pptx]

## Slide 1
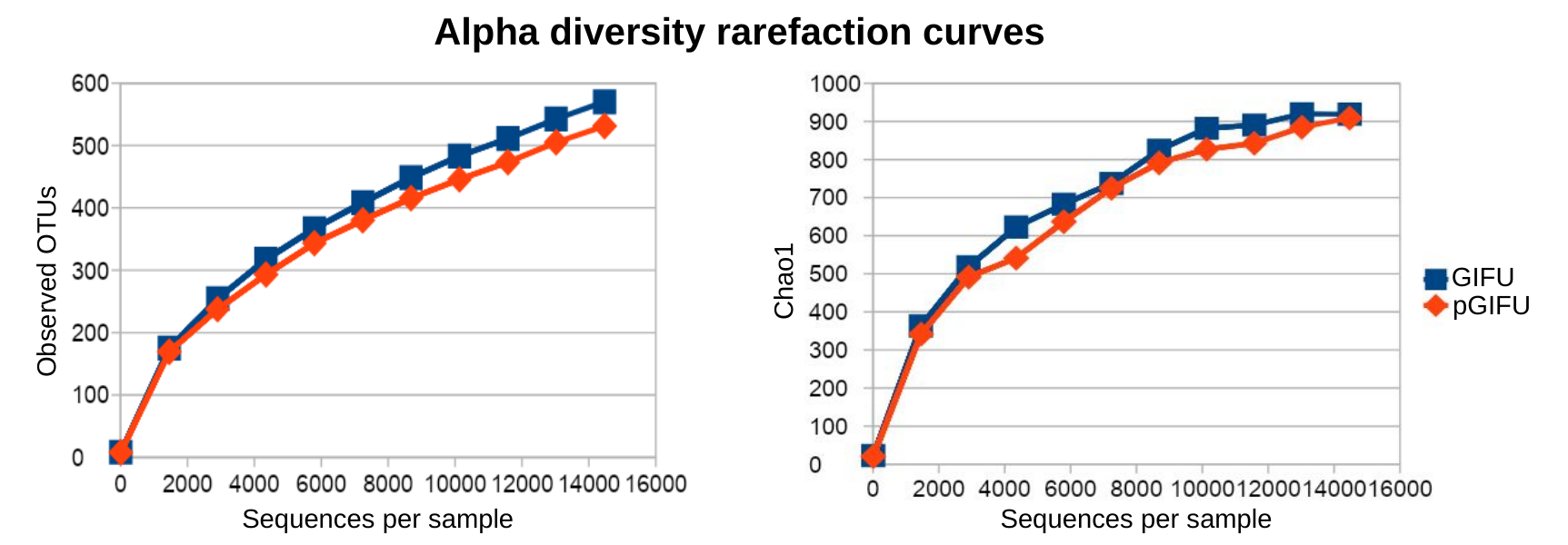

Alpha diversity rarefaction curves
GIFU
Observed OTUs
Chao1
pGIFU
Sequences per sample
Sequences per sample
