# Supplementary material for: An in vitro intestinal model captures immunomodulatory properties of the microbiota in inflammation
Source: Gut Microbes. 2022 Mar 22;14(1):2039002. doi: 10.1080/19490976.2022.2039002 (PMC8942420; doi:10.1080/19490976.2022.2039002)
Supplement: Supplemental Material [file KGMI_A_2039002_SM4653.zip › supplementary/Supplemental Fig 3.pptx]

## Slide 1
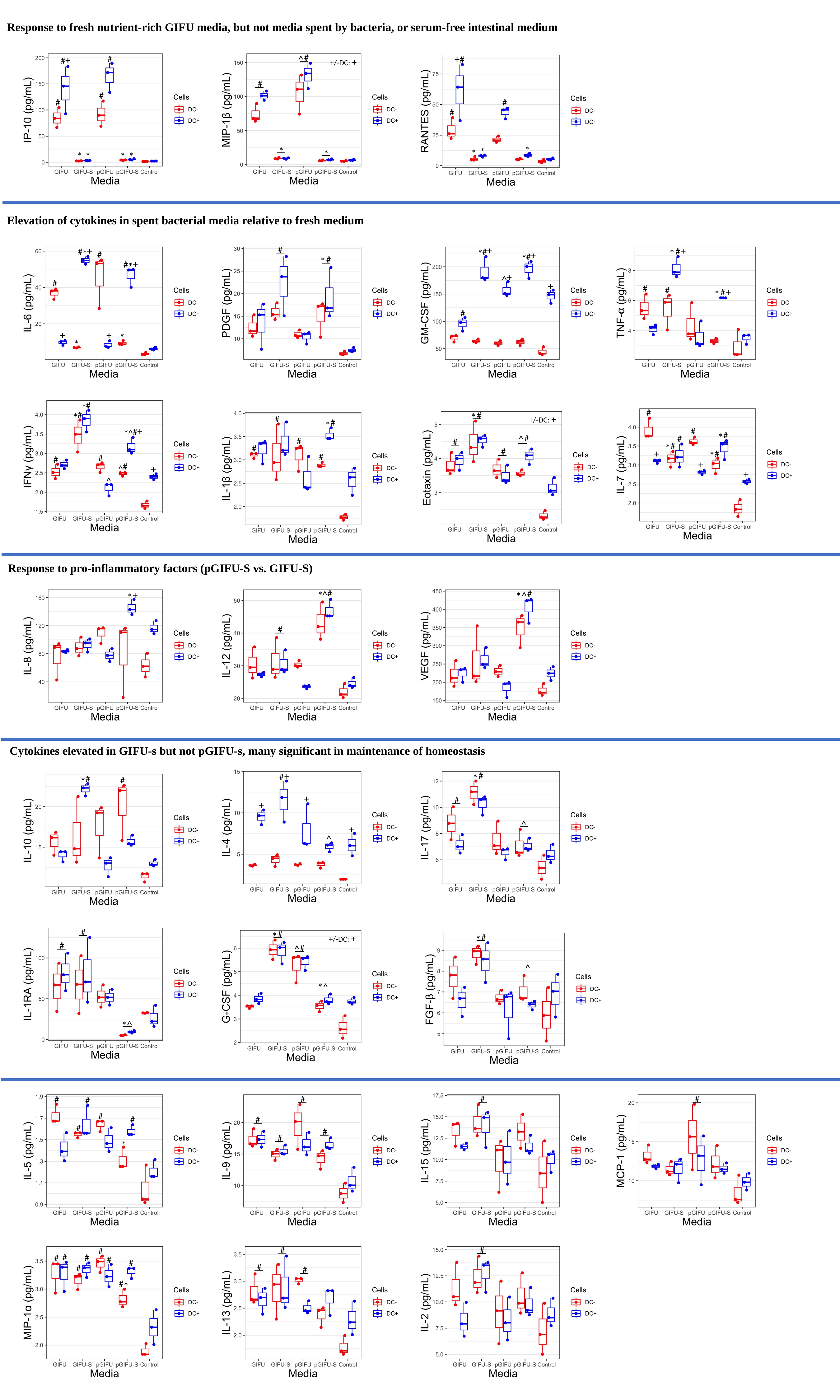

Response to fresh nutrient-rich GIFU media, but not media spent by bacteria, or serum-free intestinal medium
#
^
+
#
#
+
#
+/-DC: +
#
#
#
#
#
*
*
*
*
*
*
*
*
*
Elevation of cytokines in spent bacterial media relative to fresh medium
+
#
+
+
#
#
#
*
*
*
#
+
#
*
#
*
+
#
*
+
^
#
+
#
#
+
#
*
#
+
+
*
*
#
*
#
#
#
*
#
^
#
#
*
#
+/-DC: +
#
*
+
#
#
^
*
#
*
#
#
#
*
#
+
#
*
#
#
#
+
#
+
^
+
^
Response to pro-inflammatory factors (pGIFU-S vs. GIFU-S)
#
#
^
+
*
^
*
*
#
Cytokines elevated in GIFU-s but not pGIFU-s, many significant in maintenance of homeostasis
#
+
#
*
#
#
*
+
#
+
^
+
^
#
#
*
#
*
^
+/-DC: +
#
#
^
^
*
^
*
#
#
#
#
#
#
#
#
#
#
#
*
#
#
#
#
#
#
#
#
#
#
#
#
*
