# Supplementary figures and images for: An in vitro intestinal model captures immunomodulatory properties of the microbiota in inflammation
Source: Gut Microbes. 2022 Mar 22;14(1):2039002. doi: 10.1080/19490976.2022.2039002 (PMC8942420; doi:10.1080/19490976.2022.2039002)

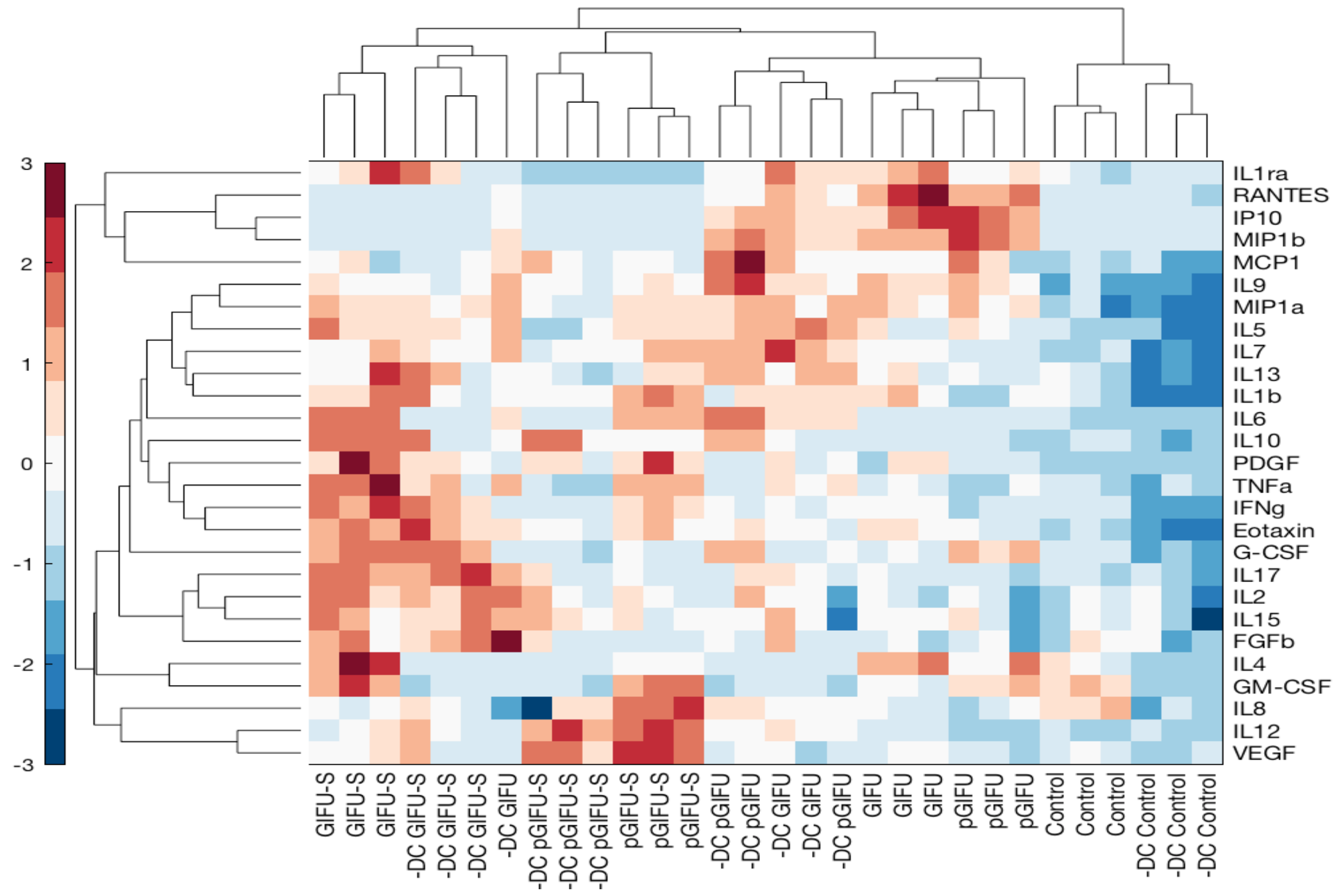

Supplement: Supplemental Material [file KGMI_A_2039002_SM4653.zip › supplementary/downloadFromZipFile 3.pdf]

**A****+DC**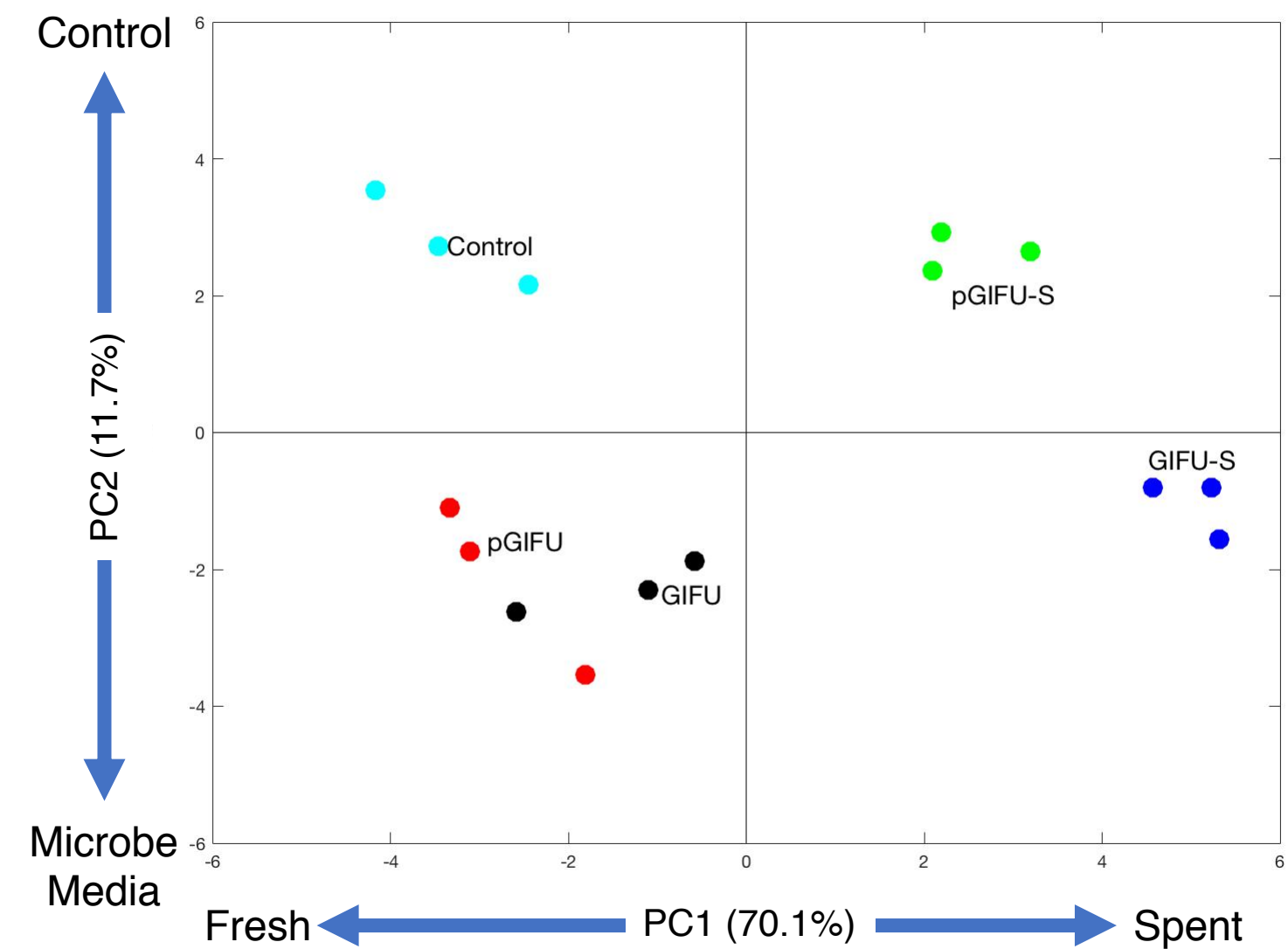**B****-DC**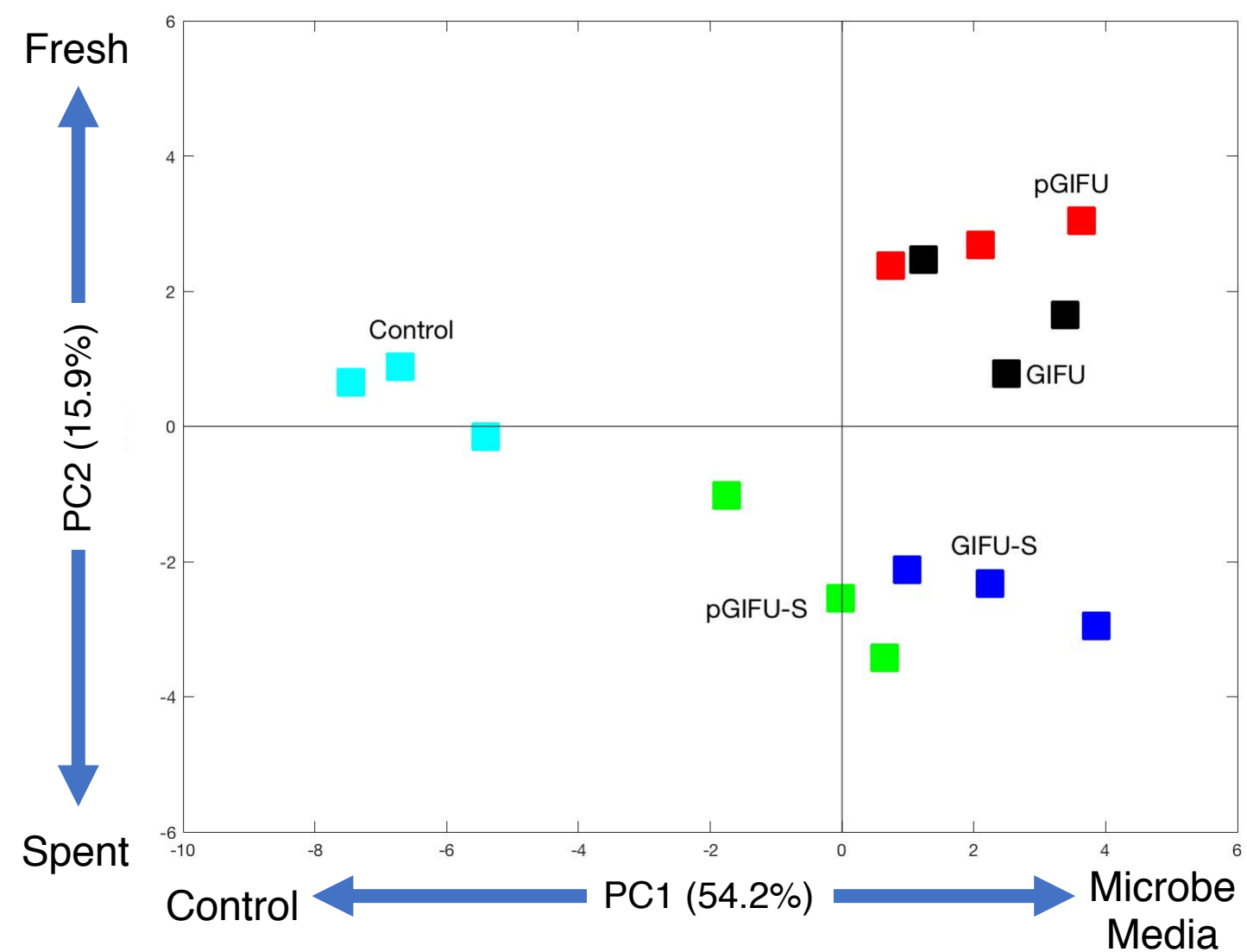**C**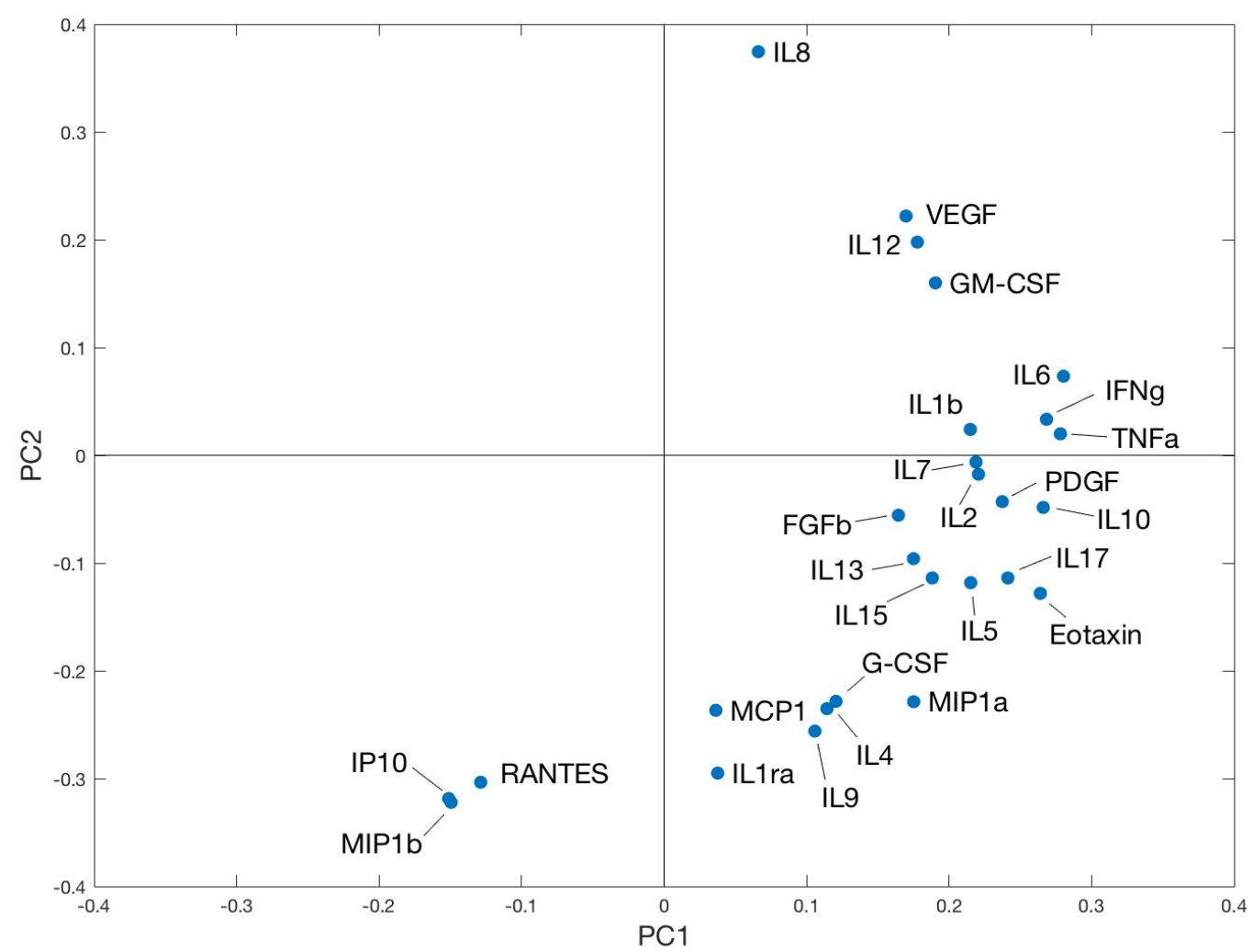**D**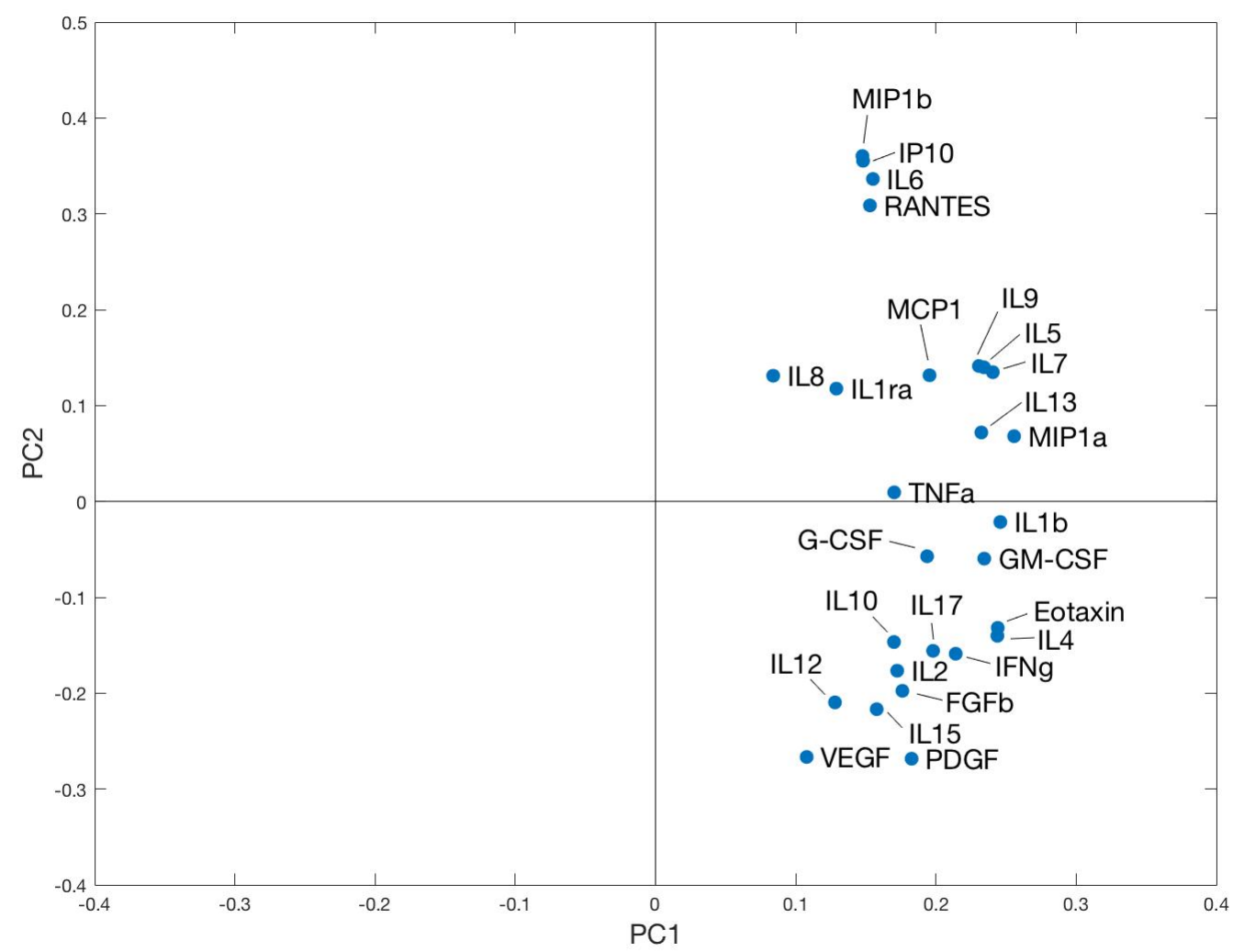

Supplement: Supplemental Material [file KGMI_A_2039002_SM4653.zip › supplementary/downloadFromZipFile 4.pdf]

**A**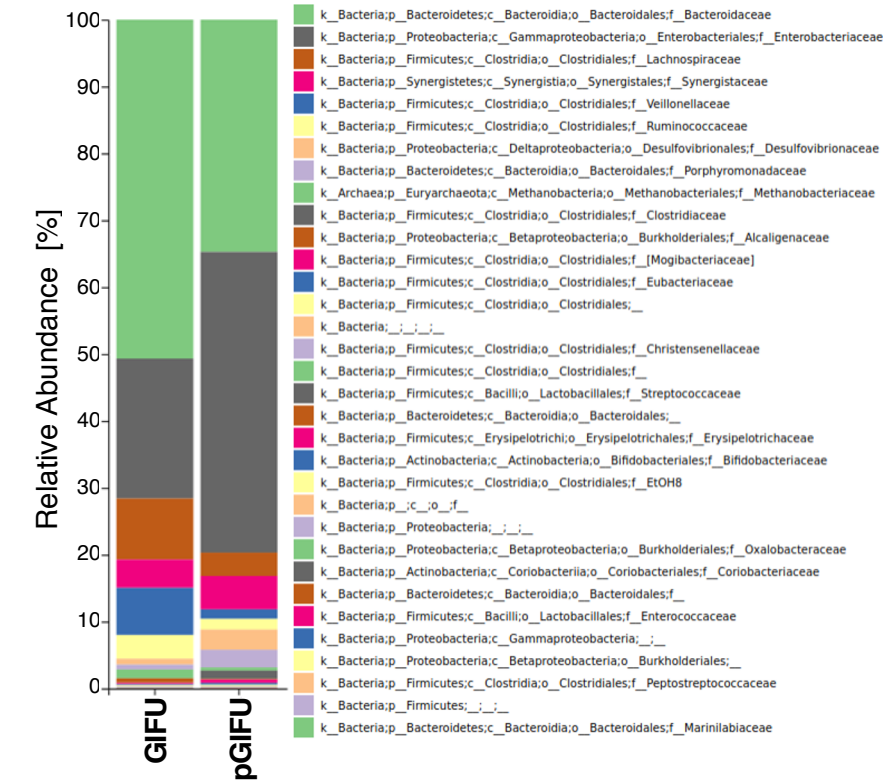**B**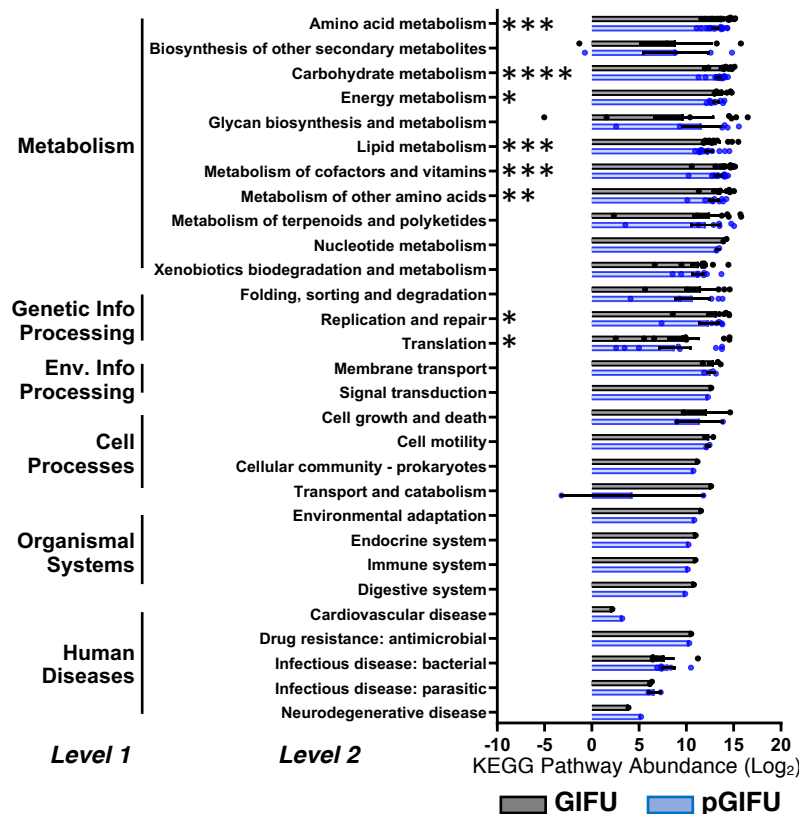**C**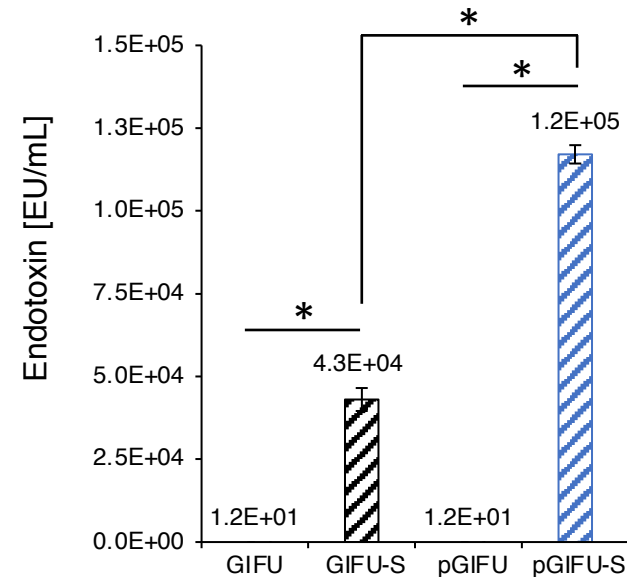

Supplement: Supplemental Material [file KGMI_A_2039002_SM4653.zip › supplementary/downloadFromZipFile.pdf]

GIFU

GIFU-S

pGIFU

pGIFU-S

Control

Zo-1

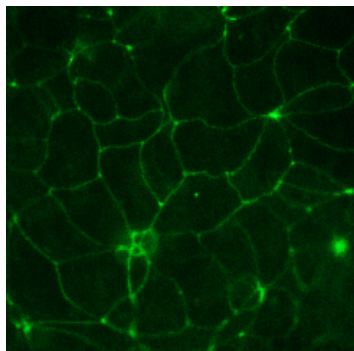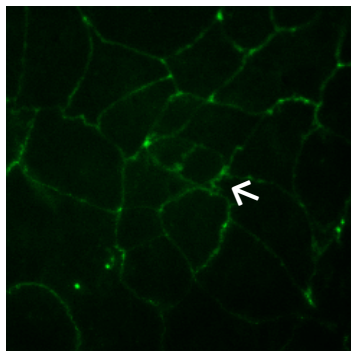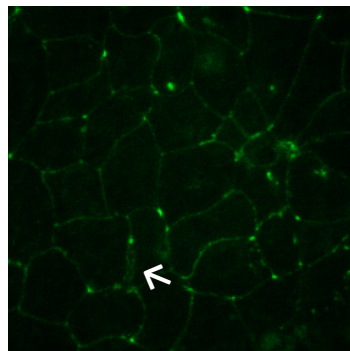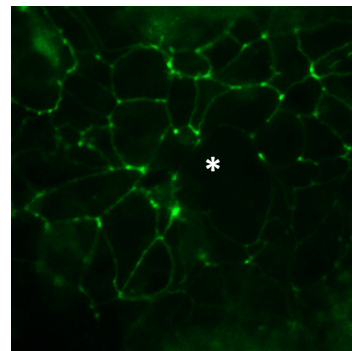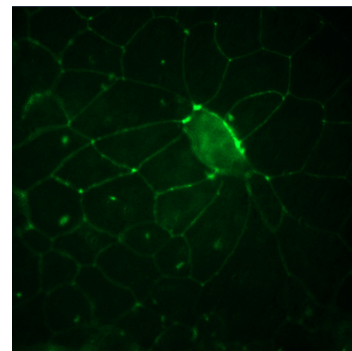

DAPI

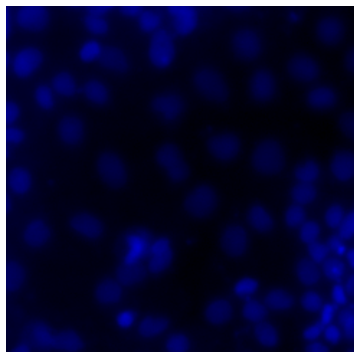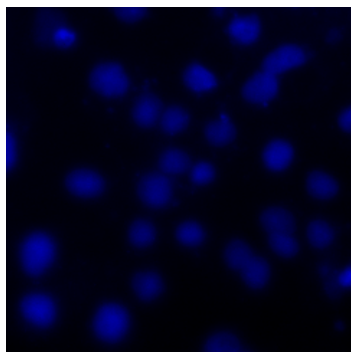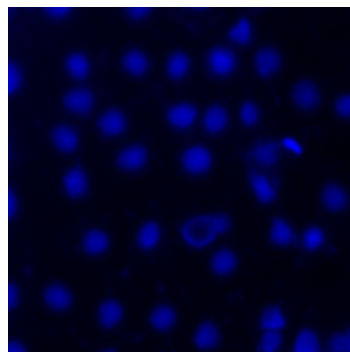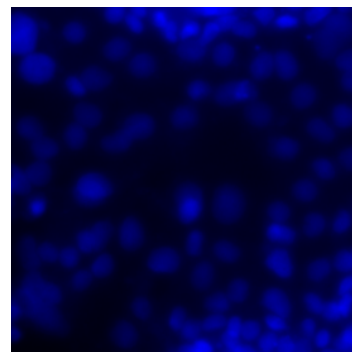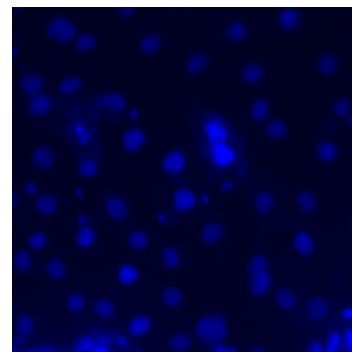

Merge

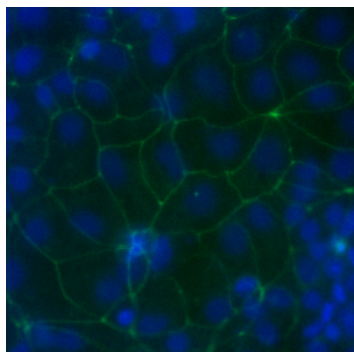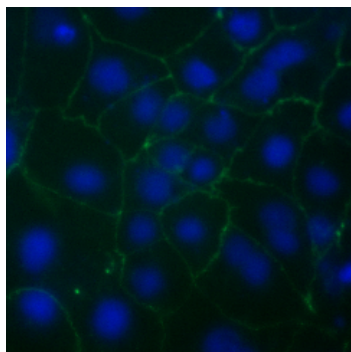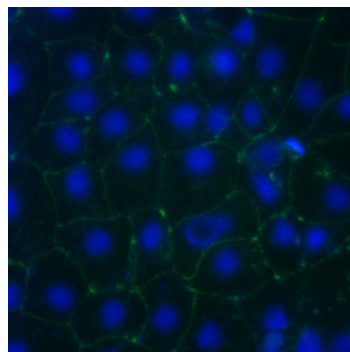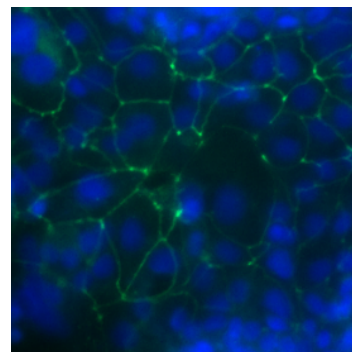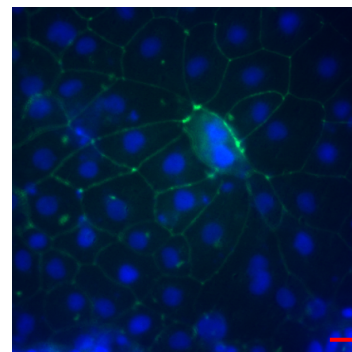

Supplement: Supplemental Material [file KGMI_A_2039002_SM4653.zip › supplementary/downloadFromZipFile2.pdf]

## Slide 1
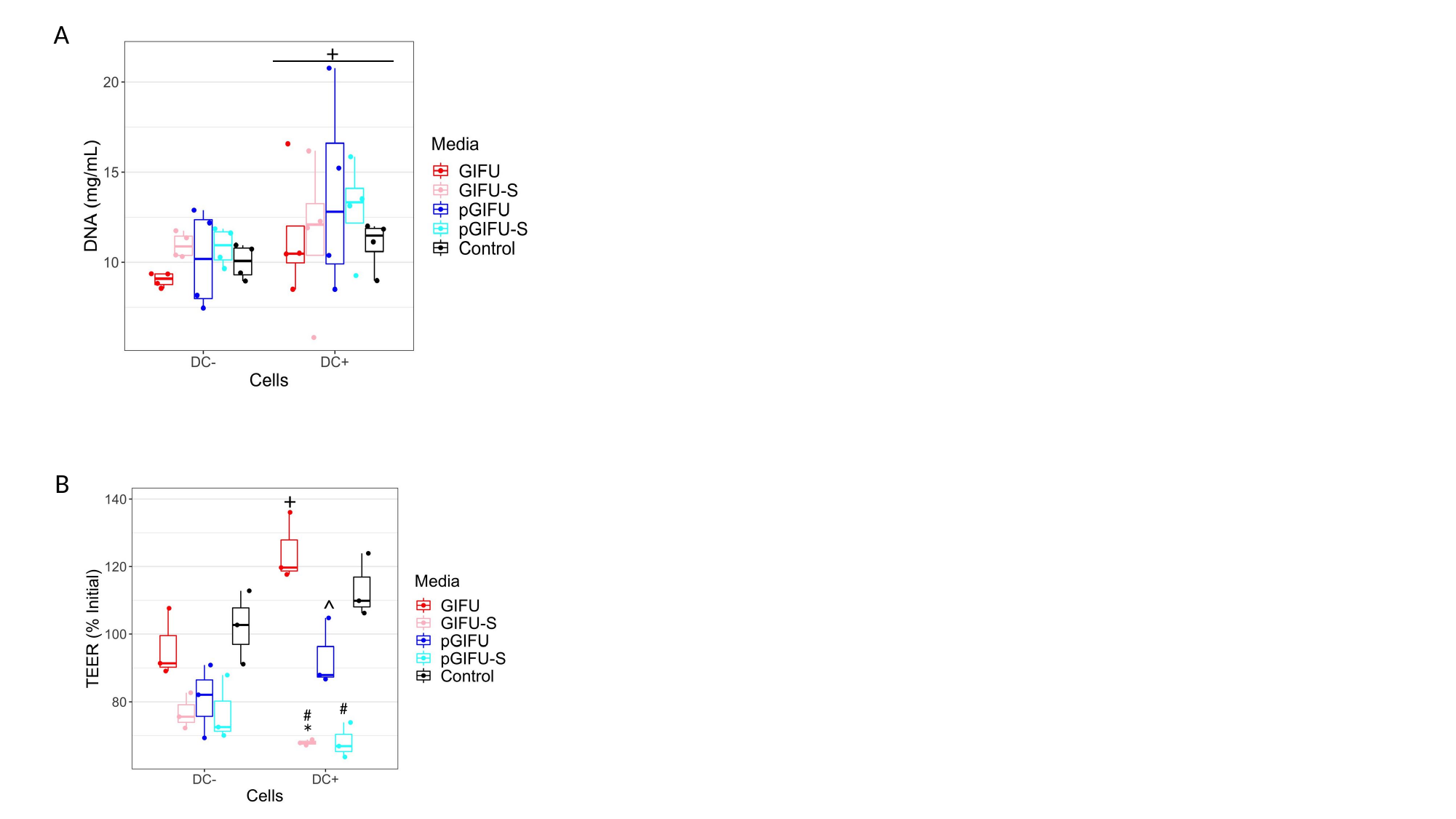

A
+
B
+
^
#
#
*

Supplement: Supplemental Material [file KGMI_A_2039002_SM4653.zip › supplementary/Fig 2.pptx]

## Slide 1
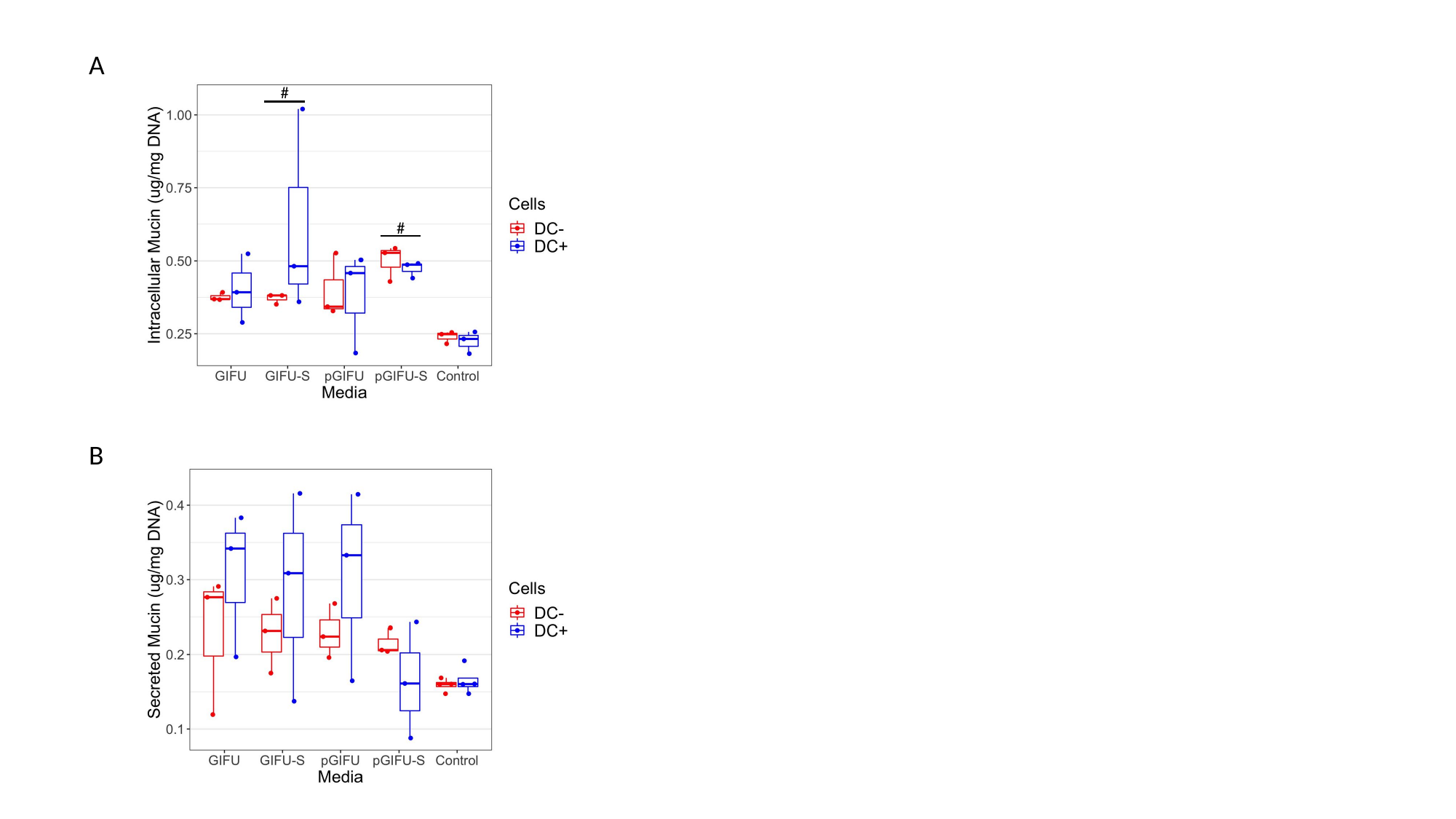

A
#
#
B

Supplement: Supplemental Material [file KGMI_A_2039002_SM4653.zip › supplementary/Fig 4.pptx]
